# In Silico Approach to Molecular Profiling of the Transition from Ovarian Epithelial Cells to Low-Grade Serous Ovarian Tumors for Targeted Therapeutic Insights

**DOI:** 10.3390/cimb46030117

**Published:** 2024-02-26

**Authors:** Asim Leblebici, Ceren Sancar, Bahar Tercan, Zerrin Isik, Mehmet Emin Arayici, Ender Berat Ellidokuz, Yasemin Basbinar, Nuri Yildirim

**Affiliations:** 1 Department of Translational Oncology, Institute of Health Sciences, Dokuz Eylul University, 35340 Izmir, Turkey; asim.leblebici@gmail.com; 2 Department of Gynecology and Obstetrics, Faculty of Medicine, Ege University, 35340 Izmir, Turkey; cerensancar@gmail.com; 3 Institute for Systems Biology, Seattle, WA 98109, USA; bahar.tercan@isbscience.org; 4 Department of Computer Engineering, Faculty of Engineering, Dokuz Eylul University, 35340 Izmir, Turkey; zerrin@cs.deu.edu.tr; 5 Department of Public Health, Faculty of Medicine, Dokuz Eylul University, 35340 Izmir, Turkey; mehmet.e.arayici@gmail.com; 6 Department of Internal Medicine, Faculty of Medicine, Dokuz Eylul University, 35340 Izmir, Turkey; enderellidokuz@hotmail.com; 7 Department of Translational Oncology, Institute of Oncology, Dokuz Eylul University, 35340 Izmir, Turkey; ybaskin65@gmail.com

**Keywords:** low-grade serous ovarian cancer, borderline, gene coexpression network, in silico integrative data analysis

## Abstract

This paper aims to elucidate the differentially coexpressed genes, their potential mechanisms, and possible drug targets in low-grade invasive serous ovarian carcinoma (LGSC) in terms of the biologic continuity of normal, borderline, and malignant LGSC. We performed a bioinformatics analysis, integrating datasets generated using the GPL570 platform from different studies from the GEO database to identify changes in this transition, gene expression, drug targets, and their relationships with tumor microenvironmental characteristics. In the transition from ovarian epithelial cells to the serous borderline, the FGFR3 gene in the “Estrogen Response Late” pathway, the ITGB2 gene in the “Cell Adhesion Molecule”, the CD74 gene in the “Regulation of Cell Migration”, and the IGF1 gene in the “Xenobiotic Metabolism” pathway were upregulated in the transition from borderline to LGSC. The ERBB4 gene in “Proteoglycan in Cancer”, the AR gene in “Pathways in Cancer” and “Estrogen Response Early” pathways, were upregulated in the transition from ovarian epithelial cells to LGSC. In addition, SPP1 and ITGB2 genes were correlated with macrophage infiltration in the LGSC group. This research provides a valuable framework for the development of personalized therapeutic approaches in the context of LGSC, with the aim of improving patient outcomes and quality of life. Furthermore, the main goal of the current study is a preliminary study designed to generate in silico inferences, and it is also important to note that subsequent in vitro and in vivo studies will be necessary to confirm the results before considering these results as fully reliable.

## 1. Introduction

Borderline ovarian tumors exhibit moderate nuclear atypia and modestly elevated mitotic activity, placing them in an intermediate state between benign and malignant tumors. It is a well-known fact that differentiating them from ovarian carcinomas is the lack of stromal invasion and rapid infiltrative development. The majority of borderline ovarian tumors (65–70%) exhibit serous histology; however, they only account for 15–20% of all serous ovarian tumors [1]. Surgery is the standard therapy for borderline ovarian tumor treatment.

Patients diagnosed with borderline ovarian tumors and exhibiting peritoneal dissemination, lymph node involvement, or those with invasive implants have a higher risk of recurrence and progression to low-grade invasive serous carcinoma (LGSC) [2]. LGSC is an invasive carcinoma that can be differentiated from serous borderline neoplasms by the presence of destructive stromal invasion. LGSC is distinguished from high-grade serous carcinoma HGSC by its lower mitotic activity. Similar to HGSC, LGSC is commonly detected at an advanced stage, resulting in a poor long-term prognosis. Nevertheless, these neoplasms exhibit different biological characteristics compared to HGSC and have a low growth rate, making them less responsive to platinum-based treatment [3].

Numerous molecular analysis studies have demonstrated that the mitogen-activated protein kinase (MAPK) pathway is commonly altered in LGSC. Among the detected mutations, KRAS, NRAS, and BRAF mutations are the most frequently observed, while TP53 mutations, which are characteristic of HGSC, are either absent or infrequent [4].

In some examples of bioinformatics methods used in borderline serous and low serous ovarian cancer research, whole-exome sequencing identified novel candidate driver genes in low-grade serous ovarian cancer [5]. Also, RNA sequencing revealed distinct molecular subtypes of borderline serous ovarian tumors [6].

This current research involved the analysis of nine GEO datasets, focusing on gene-expression data in different groups of ovarian cancer. Data preprocessing, coexpression network and differential expression analysis, and gene-set enrichment led to the identification of key genes and pathways related to low serous ovarian cancer progression, along with potential drug-gene interactions. In addition, drug response data of potential target genes in cancer cell lines and correlations of these genes with tumor microenvironment were evaluated to reveal a holistic approach. In summary, this study offers a valuable framework for personalized ovarian cancer treatments, emphasizing the importance of molecular profiling in advancing precision medicine in oncology.

In this paper, it was aimed to elucidate the molecular differences among normal ovarian epithelial cells, borderline serous ovarian tumors, and low-grade serous ovarian tumors in order to establish a basis for targeted therapy strategies.

## 2. Materials and Methods

### 2.1. Dataset Selection

**Figure 1:**
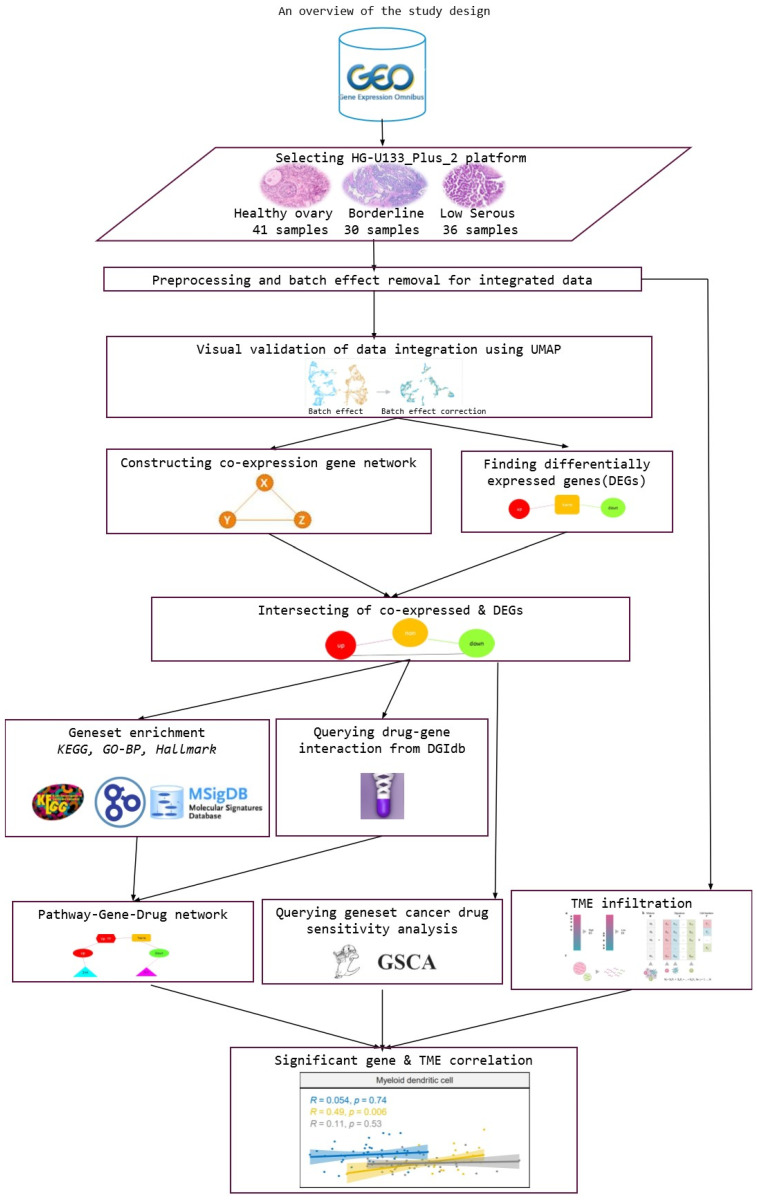
An overview of the study design.

We downloaded nine gene-expression datasets from the Gene Expression Omnibus database (GEO http://www.ncbi.nlm.nih.gov/geo/ accessed on 15 December 2023) with the filters Study type = ‘Expression profiling by array’, Organisms = ‘Homo sapiens’, and Platform = ‘GPL570 ([HG-U133_Plus_2] Affymetrix Human Genome U133 Plus 2.0 Array)’. We selected datasets containing normal, borderline, and low serous samples (Table 1). Figure 1 provides a visual representation of the study design, offering an extensive overview of the key elements and methodologies used in the research.

The datasets we have analyzed are listed below.

The GSE18520 study utilizes whole-genome oligonucleotide arrays to conduct expression profiling on microdissected late-stage, high-grade papillary serous ovarian adenocarcinomas, identifying and validating a prognostic gene signature associated with survival and uncovering novel survival factors in ovarian cancer. The study includes 63 samples with healthy and advanced ovarian tumors.

The GSE27651 study investigates the molecular progression from serous borderline ovarian tumors (SBOT) to LGSC and HGSC, identifying the upregulation of the anterior gradient homolog 3 (AGR3) gene in SBOT, suggesting its potential role as a prognostic marker for improved survival in both LGSC and HGSC. The study includes 49 samples with healthy, borderline, LGSC, and HGSC.

The GSE14001 study identifies and validates higher PAX2 expression in ovarian tumors of low-malignant potential (LMP) and low-grade serous carcinomas compared to high-grade serous carcinomas, supporting the two-tiered hypothesis of distinct tumorigenic pathways, and suggesting PAX2 as a potential biomarker and therapeutic target for individualizing chemotherapy in ovarian LMP tumors and LGSC. The study includes 23 samples with healthy, LGSC, and HGSC.

The GSE27659 study investigates the genetic features of advanced-stage, low-grade ovarian serous carcinomas, finding that those with and without adjacent serous borderline tumors share similar loss of heterozygosity patterns. While TP53 mutations are absent, BRAF mutations are rare in aggressive LGSC, suggesting their potential derivation from serous borderline tumors without BRAF mutation, with patients harboring BRAF or KRAS mutations displaying a better clinical outcome. The study includes 91 samples with healthy, borderline, and LGSC.

The GSE73091 study investigates the gene-expression profiles of LGSC cancer compared to HGSC, revealing distinct molecular alterations during LGSC progression by analyzing nine magnetically sorted epithelial tumor samples from matched primary tumors, ascites, and metastases. The study includes 9 samples with LGSC, ascite, and HGSC.

The GSE9891 study employed microarrays to analyze the expression levels of 285 ovarian samples from the Australian Ovarian Cancer Study (AOCS) on the Affymetrix U133 Plus 2.0 platform (GPL570), aiming to identify novel molecular subtypes of ovarian tumors through disease-state analysis.

The GSE14407 study utilized gene-expression profiling to compare 12 healthy ovarian surface epithelial cells with 12 laser-captured microdissected serous papillary ovarian cancer cells, revealing over 2000 significantly differentially expressed genes, implicating key signaling pathways and suggesting that ovarian surface epithelial cells function as an adult stem-cell niche, with the deregulation of genes associated with maintaining quiescence playing a crucial role in the initiation and development of ovarian cancer. The study includes 24 samples with healthy and serous ovarian tumors.

The GSE36668 study identifies and validates 21 mRNAs differentially expressed between moderately/poorly differentiated serous ovarian carcinomas (MD/PD SC), serous ovarian borderline tumors (SBOT), and superficial scrapings from normal ovaries (SNO), revealing significant correlations with clinical parameters, such as VEGFA and ZNF385B correlating with survival and FOXM1 and TPX2 correlating with the normalization of serum CA125, providing insights into potential molecular pathways, including VEGFA, FOXM1, TPX2, BIRC5, and TOP2A, implicated in the tumorigenesis of MD/PD SC. The study included 12 samples with healthy, borderline, and serous ovarian tumors.

The GSE54388 study aims to explore the transcription factors associated with the pathogenesis of HGSC utilizing transcriptome profiling on laser microdissected epithelial tumor samples from ovarian cancer patients and ovarian surface epithelium (HOSE) samples, with the Affymetrix human genome U133 Plus 2.0 microarray. The study included 22 samples with healthy and HGSC.

### 2.2. Microarray Gene-Expression Data Preprocessing

We normalized microarray data using the robust multiarray average (RMA) approach. Gene probe names were converted to EntrezID, and we merged the datasets. The results of the normalization of the datasets are summarized in Supplementary Figures S1–S9.

### 2.3. Data Aggregation and Data Integration with Batch-Effect Correction

We aggregated multiple probe values mapped to the same EntrezID using median values. We did batch-effect correction using the combat method, since the data had been collected by different labs.

### 2.4. Coexpression Network Analysis

Weighted gene-coexpression network analysis was performed using the WGCNA R package (1.71-1) [7]. The aim of this analysis is to extract gene-expression modules that have the highest correlation with phenotypes, namely normal ovary, borderline serous, and low serous ovarian cancer. Initially, a soft threshold power was chosen with a scale-free topology criterion, and, with this threshold power, a weighted gene adjacency matrix representing a gene-coexpression network was constructed, where each link shows the coexpression similarity between gene pairs. The adjacency matrix was transformed into a topological overlap matrix (TOM) to minimize the effects of noise, and the dissimilarity matrix was computed as 1-TOM. The hierarchical clustering of the dissimilarity matrix was used to identify the modules in the network and a dendrogram was constructed. Modules were identified where the dendrogram was cut. We used a standard method to cut the branches using the “dynamicTreeCut” package (1.63-1). Dynamic tree cutting was used to identify modules with highly similar gene-expression profiles. Highly coexpressed modules were merged.

### 2.5. Differentially Expressed Genes Analysis

We used differential gene-expression (DGE) analysis to identify genes that are differently expressed between different groups of samples. This helps us identify genes that may be involved in specific biological or clinical conditions. In this study, we used DGE analysis to compare the gene expression between healthy ovarian tissue, borderline serous ovarian tumor tissue, and low-grade serous ovarian cancer tissue. We used two methods to identify differentially expressed genes: limma (linear models for microarray data) method followed by *p*-value adjustment (Benjamini–Hochberg method) and fold change. We identified significant differentially expressed genes by filtering for the genes with an absolute log2 fold-change value greater than 1.0 and adjusted *p*-value less than 0.05, then intersected these genes with the genes that were identified as important (correlation value > 0.3) in the WGCNA modules between phenotype and gene-expression profiles. This allowed us to identify genes that are both differentially expressed and important for ovarian cancer.

### 2.6. Gene-Set Enrichment Analysis

Gene-set enrichment analyses were performed utilizing KEGG, GO-BP, Cancer Hallmark, and TRRUST Transcription Factors databases with the enrichR [8] package. We identified significant pathway terms by filtering out terms with adjusted *p*-values less than 0.05.

### 2.7. Drug–Gene Interaction Analysis

We queried the Drug–Gene Interaction Database (DGIdb) for drugs that could bind to target genes that we identified at the intersection of coexpression network modules and DEGs [9]. We selected inhibitors for the overexpressed genes and activators for the underexpressed genes.

### 2.8. Visualization of the Integrated Pathway Results

We integrated the pathways associated with significantly overexpressed and underexpressed genes as well as drug targets and subsequently visualized them using Cytoscape via the RCy3 package (2.22.1) [10].

### 2.9. Estimation of Tumor-Microenvironment Infiltration

The microenvironment cell populations counter (MCP-counter) method was used to estimate the amount of immune and stromal cell populations infiltrating the tissue [11]. T-cell, T-cell CD8+, cytotoxicity score, NK cell, B cell, macrophage–monocyte, myeloid dendritic cell, neutrophil, endothelial cell, and cancer-associated fibroblast abundances were estimated as the amount of immune and stromal cell populations infiltrating the tissue. We compared cell-type abundances in samples from different phenotypes using the Kruskal–Wallis test.

### 2.10. Drug Sensitivity Analysis of Potentially Important Genes

We employed the Gene Set Cancer Analysis (GSCA) [12] web tool, which retrieves results from the Genomics of Drug Sensitivity in Cancer (GDSC) database and Cancer Therapeutics Response Portal (CTRP), to correlate the drug-response data acquired for the characterized human cancer cell lines associated with our coexpressed and DEGs.

### 2.11. Software Environment and Packages

We listed the packages/tools and the analysis we performed using them in Table 2.

## 3. Results

### 3.1. Batch-Effect Removal

**Figure 2:**
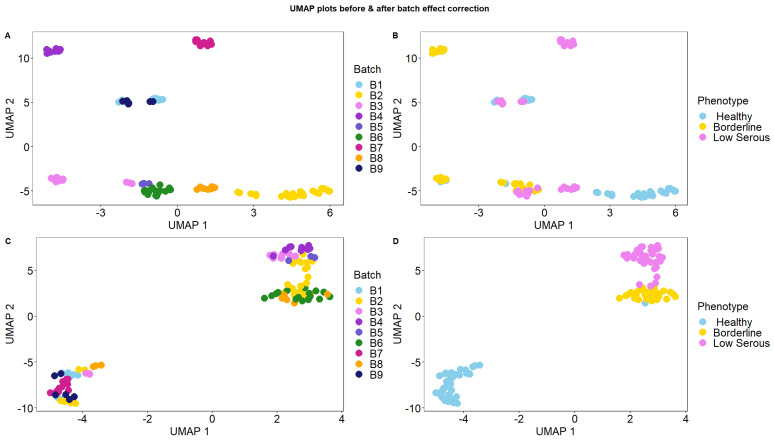
(**A**,**B**) UMAP plots showing the nine datasets before batch-effect removal colored by batches and phenotypes, respectively. (**C**,**D**) UMAP plots showing the nine datasets after batch-effect removal colored by batches and phenotypes respectively.

We performed batch-effect removal on the datasets downloaded from the GEO database (Table 1) and merged the datasets. Figure 2 shows the UMAP plots labeled by the phenotypes and batches before and after removing the batch effects. Before batch-effect removal, the sample batches drove the clustering; after batch correction, the samples were clustered based on their phenotypes (healthy, borderline, and low serous) (Figure 2).

### 3.2. Differential Gene-Expression Analysis

Upon comparing the healthy and borderline groups, 1640 differentially expressed genes were detected, 765 were downregulated and 875 were upregulated in the borderline compared to healthy. When the borderline and low serous groups were compared, we observed that, of 1098 differentially expressed genes, 749 were downregulated and 349 were upregulated in the low serous compared to the borderline. When the healthy and low serous groups were compared, we found that, of 2158 differentially expressed genes, 1192 were downregulated and 966 were upregulated in the low serous compared to the healthy (Supplementary Sheets S1–S3).

When we intersected the DEGs between the healthy and borderline groups with the WGCNA modules that we selected in relation to the Healthy(−) vs. Borderline(+) and Healthy(+) vs. Borderline(−), 629 genes and 679 genes were found to be in the module and DEGs intersection, respectively.

Likewise, when we intersected the DEGs between the borderline and low serous groups with the WGCNA modules that we selected in relation to Borderline(−) vs. Low serous(+) and Borderline(+) vs. Low serous(−) related modules, 120 genes and 400 genes were found to be in the module and DEGs intersection, respectively.

When we compared the DEGs between the healthy and low serous groups with the WGCNA modules that we selected in relation to Healthy(−) vs. Low serous(+) and Healthy(+) vs. Low serous(−) related modules, 914 genes and 562 genes were found to be in the module and DEGs intersection, respectively (Table 3).

### 3.3. Gene-Coexpression Network Analysis

Weighted gene-coexpression network analysis (WGCNA), identifies groups of coexpressed genes in high dimensional datasets, helping uncover functional relationships. Undirected correlation measures the strength of relationships between genes without implying causality. WGCNA utilizes undirected correlations to construct gene-coexpression networks, revealing interconnected gene clusters with shared functions. WGCNA constructs a gene-coexpression network in accordance with the scale-free topology criterion [7]. As shown in Figure S10, the soft threshold value “8” was selected where the scale-free topology fit index curve reached the lowest value, indicating R^2^ > 0.8 [7].

**Figure 3:**
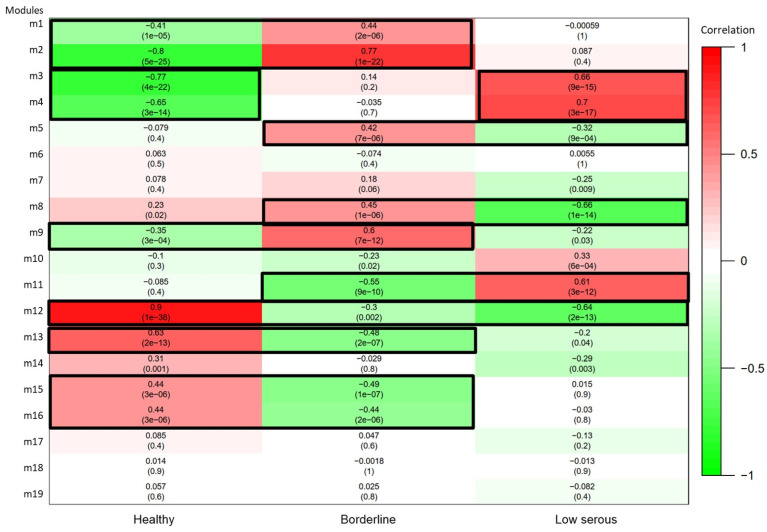
A module-trait heatmap is a visual representation of the correlations between the expression levels of genes in each module and different clinical and pathological traits. The heatmap is generated by calculating the Pearson correlation coefficient (PCC), also known as the eigengene significance, between the expression levels of genes in each module and each trait. The PCCs are then color coded, with red representing positive correlations and green representing negative correlations. The darker the color, the stronger the correlation. (Each box displays a correlation value above and a corresponding *p*-value in parentheses below.).

The correlations (together with *p* values) between WGCNA modules and phenotypes are shown in Figure 3. Dynamic tree cutting was used to identify modules with similar gene-expression profiles. Similar modules were combined according to a height cutoff threshold of 0.25, and 19 modules were obtained.

WGCNA helps prioritize groups of genes with potential functional significance, while DGE analysis helps identify specific genes within these groups that are critical for understanding the observed changes in gene expression between different conditions. Together, they offer a comprehensive view of the gene-expression data and its biological implications. We selected modules with a correlation value > 0.3 between phenotype and gene-expression profiles.

### 3.4. Gene-Set Enrichment and Drug–Gene Interaction Analysis

**Figure 4:**
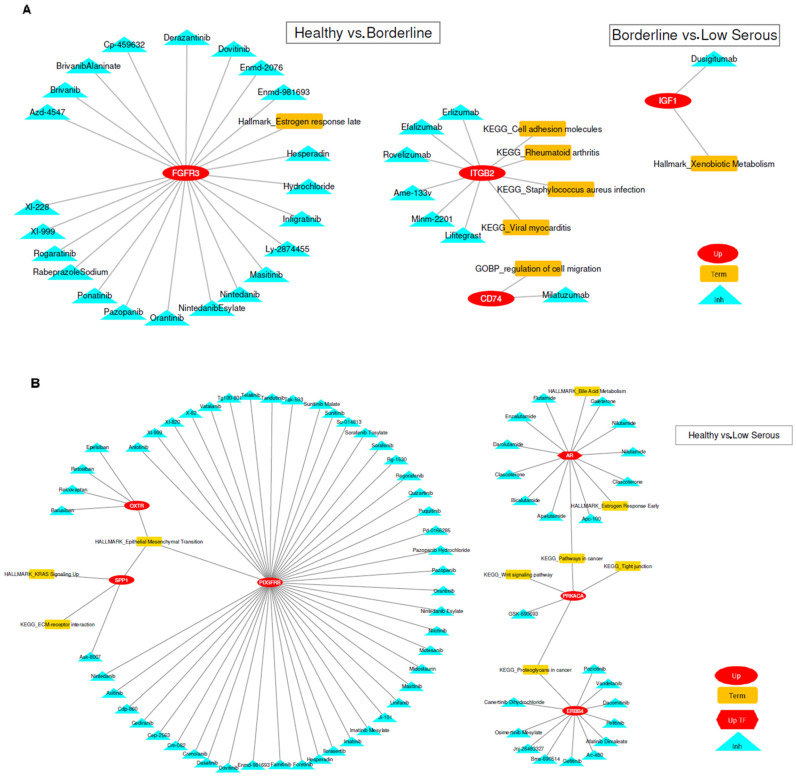
(**A**) Healthy vs. borderline and borderline vs. low serous groups pathway term gene–drug interaction results plot. (**B**) Healthy vs. low serous group pathway term gene and transcription factor gene–drug interaction results plot. (Abbreviations of figure legend, Up: upregulated gene, Up-TF: upregulated transcription factor gene, Inh: inhibitory drug).

We performed gene-enrichment analysis using the genes at the intersection of coexpression and differential expression analysis. Utilizing KEGG, Gene Ontology-Biological Processes, Cancer Hallmark, and TRRUST Transcription Factors databases, we considered pathways with an adjusted *p*-value less than 0.05 as significant (Supplementary Sheets S4–S11). For genes in significant pathways, we searched the DGIdb database for drug–gene interactions using the rDGIdb package, retrieving results from more than thirty reliable sources. For upregulated genes, we used the interaction types (“antagonist”, “antibody”, “antisense oligonucleotide”, “blocker”, “cleavage”, “inhibitor”, “inhibitory allosteric modulator”, “inverse agonist”, “negative modulator”, “partial antagonist”, and “suppressor”) to find inhibitory drug interactions. For downregulated genes, we used the interaction types (“activator”, “agonist”, “chaperone”, “cofactor”, “inducer”, “partial agonist”, “positive modulator”, “stimulator”, and “vaccine”) to find activator drug interactions. We provide Table 4 and Table 5, where coexpressed genes, differentially expressed genes, gene-set enrichment, and drug-interaction analysis results can be analyzed together. Additionally, the associated results are visualized and summarized in Figure 4A,B.

### 3.5. Gene-Set Cancer Drug Sensitivity (GDSC) Analysis

**Figure 5:**
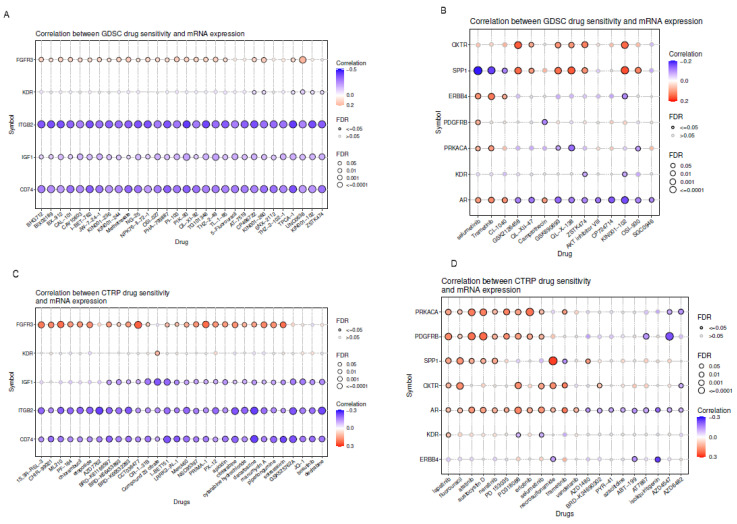
(**A**,**C**) show the correlation of drug sensitivity with gene expression for the healthy to borderline and borderline to low serous transition (IGF1) in our results from the GDSC and CTRP databases. (**B**,**D**) show the drug sensitivity results from the GDSC and CTRP databases for healthy to low serous transition genes. Purple bubbles represent higher sensitivity, while red bubbles represent lower sensitivity with increasing gene expression.

We used the GSCA tool to correlate the drug sensitivity with the expression of genes that were found to be significant in between groups and the drugs that target them. A positive correlation coefficient shows that upregulated gene expression is associated with drug resistance. We found that increased mRNA expression levels of the FGFR3 gene (indicated by red bubbles) in the transition from healthy to borderline showed resistance to drugs. Purple bubbles show the sensitivity of ITGB2 and CD74 to a group of known drugs such as Methotrexate, 5-Fluorouracil in the transition from healthy to borderline and IGF1 in the transition from borderline to low serous in Figure 5A–C. Increased SPP1 gene shows sensitivity to Selumertinib and Trametinib, increased PDGFRB gene shows sensitivity to AZD4547 for PDGFRB, and increased ERBB4 gene shows sensitivity to isoliquiritigenin in the transition from healthy to low serous (Figure 5B–D). Although drug responses are very tissue dependent, the results obtained from the GSCA query are valuable for discussion in terms of repositioning. Purple bubbles represent higher sensitivity, while red bubbles represent less sensitivity with increased gene expression.

### 3.6. Estimation of Tumor-Microenvironment Infiltration

**Figure 6:**
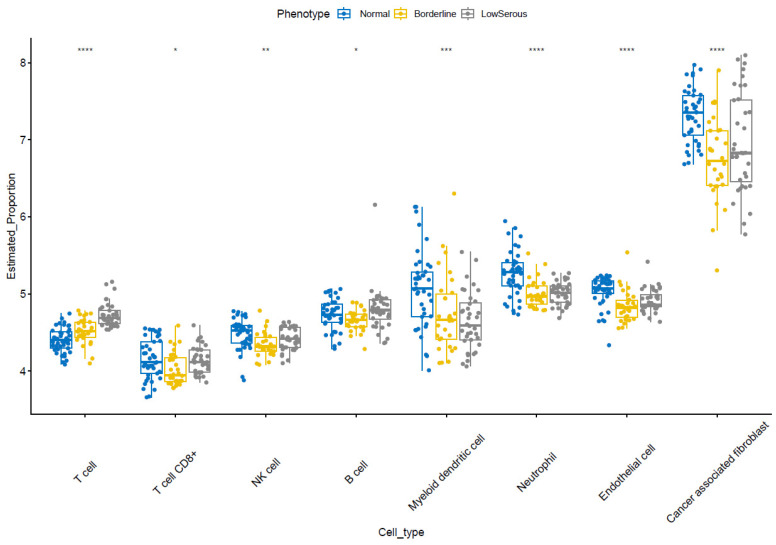
Estimation of tumor-microenvironment infiltration by MCP counter TME infiltration. (* *p* < 0.05, ** *p* < 0.01, *** *p* < 0.001, **** *p* < 0.0001).

While there was an increasing trend in T-cell population abundance in the transition from healthy tissue to low serous, this was the opposite for myeloid dendritic cells. The abundance of T-cell CD8+, NK-cell, B-cell, neutrophil, endothelial cell, and cancer-associated fibroblast populations decreased in the transition from healthy to borderline but increased in the transition from borderline to low serous Figure 6.

### 3.7. Correlation Analysis for Gene Expression and Tumor Microenvironment

**Figure 7:**
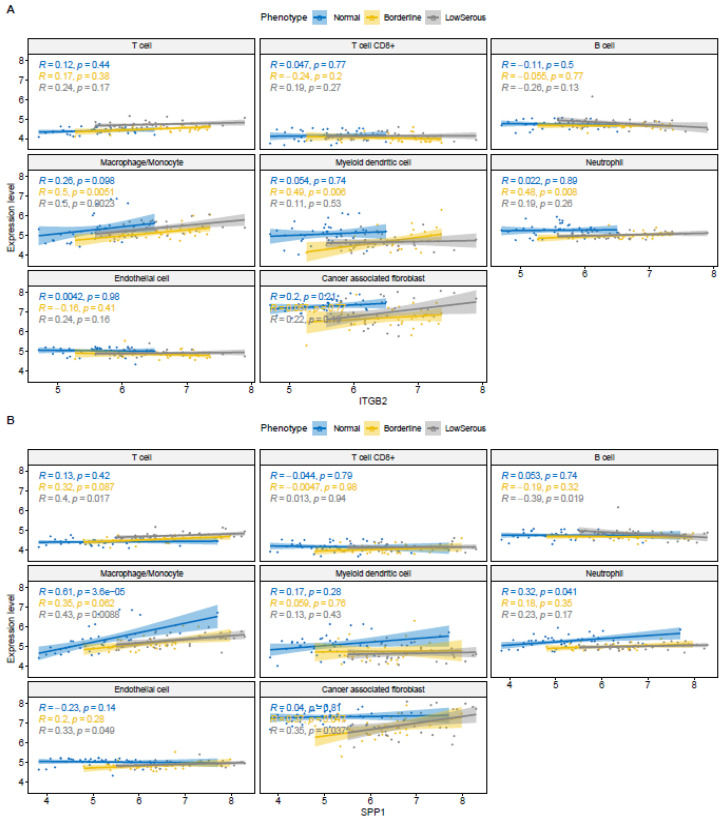
(**A**). Scatter plots with Spearman correlation between ITGB2 gene expression and TME infiltration proportion. (**B**). Scatter plots with Spearman correlation between SPP1 gene expression and TME infiltration proportions.

We found a moderate correlation between ITGB2 expression and the macrophage–monocyte proportion in borderline and low serous tissue (*r* = 0.5, *p* = 0.0051, *r* = 0.5, *p* = 0.0023, respectively), as well as a moderate correlation with myeloid dendritic cell (*r* = 0.49, *p* = 0.006) and neutrophil (*r* = 0.48, *p* = 0.008) in borderline tissue (Figure 7A). A moderate correlation between CD74 expression and B cell in borderline tissue (*r* = 0.46, *p* = 0.012), a moderate correlation between macrophage–monocyte in borderline and low serous tissue (*r* = 0.5, *p* = 0.0051, *r* = 0.5, *p* = 0.0023, respectively) was found. We also found a moderate correlation between myeloid dendritic cell (*r* = 0.49, *p* = 0.006) and neutrophil (*r* = 0.48, *p* = 0.008) in borderline tissue. For IGF1, whose expression increases in the transition from borderline to low serous, we found a moderate correlation between its expression and T cell in low serous tissue (*r* = 0.5, *p* = 0.002), and a moderate correlation between cancer-associated fibroblast in borderline and low serous tissue (*r* = 0.5, *p* = 0.0059, *r* = 0.48, *p* = 0.0036, respectively). For SPP1 in the EMT pathway, whose expression increases in the transition from healthy to low serous, there is a moderate correlation between its expression and T cell in low serous tissue (*r* = 0.4, *p* = 0.017), a moderate negative correlation between B cell in low serous tissue (*r* = −0.39, *p* = 0.019), a moderate negative correlation between macrophage–monocyte in low serous tissue (*r* = 0.43, *p* = 0.0088) and a moderate correlation between cancer-associated fibroblast in borderline and low serous tissue (*r* = 0.37, *p* = 0. 047, *r* = 0.35, *p* = 0.037, respectively) (Figure 7B). For PDGFRB, which is in the EMT pathway with increased expression in the transition from healthy to low serosa, we found a moderate correlation between its expression and cancer-associated fibroblast in borderline tissue (*r* = 0.56, *p* = 0.0016) and a high correlation in low serous (*r* = 0.75, *p* = 6.6 × 10^−7^). For ERBB4 in the proteoglycans in cancer pathway, whose expression increases in the transition from healthy to low serosa, we found a moderate correlation between its expression and myeloid dendritic cell in borderline tissue (*r* = −0.59, *p* = 7 × 10^−4^), neutrophil in borderline tissue (*r* = −0.56, *p* = 0.0017), and cancer-associated fibroblast in borderline tissue (*r* = 0.37, *p* = 0.0045). For AR gene, which is in the estrogen response early pathway with increased expression in the transition from healthy to low serosa, correlation of its expression with T cell in borderline tissue (*r* = −0.39, *p* = 0.035), with macrophages–monocyte in low serous tissue (*r* = −0.37, *p* = 0.028), with myeloid dendritic cells in borderline tissue (*r* = 0.4, *p* = 0.029), and with neutrophil (*r* = −0.49, *p* = 0.0063), we found a moderate negative correlation (Supplementary Figures S11–S20).

## 4. Discussion

Ovarian cancer is a very heterogeneous disease. The most common type is epithelial ovarian cancer, and high-grade (HG) serous tumors are the most common histology [20]. Recent data suggest that HG serous tumors mostly. originate from fallopian tube epithelium (STIC lesions) with p53 abnormality. Other rare histological subtypes, such as clear-cell and endometrioid tumors, arise from endometriotic cysts associated with endometriosis, and MOC from transitional cell nests at the tubal–mesothelial junction [21,22]. Just one histological type, low-grade (LG) serous tumors, are accepted to have a clearer progression model from benign serous cystadenoma to borderline serous tumor and then low-grade carcinoma [23,24]. Since the behavior and the prognosis of each histology differ from each other, adjuvant treatment of each case has been managed individually and translational medicine evolves the treatment modalities from “one fits for all” to targeted therapies according to molecular alterations. In this unique study, we investigated the differentially expressed genes and performed gene-coexpression network and drug–gene interaction analyses to identify the potential targeted therapies in the biologic continuum of normal ovarian epithelial cells, borderline serous ovarian tumor cells, and, finally, low-grade serous ovarian epithelial cells.

Most of the studies regarding LG serous tumors demonstrated that K-RAS and B-RAF proto-oncogene mutations are frequent, and RAS mutations were found to be associated with the recurrence of LG serous tumors [25,26,27]. Since standard chemotherapy regimens are not as effective in LG tumors as they are in HG tumors, recent studies have focused on targeted therapies related to aforementioned mutations and also hormonal therapies. Tamoxifen, letrozole, anastrozole, and fulvestrant were used in the studies as maintenance therapy after platinum and taxane chemotherapy or single-agent therapy after surgery, both in primary treatment and recurrent settings [28,29]. Gershenson et al. reported the objective response rate (ORR) for aromatase inhibitors was 13% compared with only 5.9% for tamoxifen in recurrent LG serous ovarian tumor patients [30]. In a similar phase-II study with 36 LG serous ovarian tumor patients, ORR for anastrozole was only 14% [29]. MEK inhibitors are the second group of drugs of interest for the treatment of LG serous tumors since they target RAS and RAF mutations. In a recent study, trametinib was compared with standard therapy in 260 recurrent LG serous tumor patients [31]. In this study, the median PFS was in favor of the trametinib group, 13 vs. 7.2 months (HR 0.48; 95%CI: 0.36–0.64) and ORR was 26% vs. 6%. Selumetinib is another MEK inhibitor used for LG serous tumors. In a phase-II study including 52 recurrent LG serous tumor patients, RR was 15%, and 65% of patients had stable disease [30].

Our study aims to bring new perspectives to the treatment of LG serous ovarian tumors by focusing on the molecular alterations between normal tissue, borderline, and LG serous tumors. To do so, an in silico integrated data analysis was performed to provide explanations adaptable to clinical practice, and the results were evaluated with current literature. FGFR3 (fibroblast growth factor receptor 3) is a protein-coding gene and one of the most important genes differentially expressed between normal ovarian tissue and borderline serous tumors in our study. Amplification of FGFR leads to enhanced activation of downstream signaling pathways (such as phospholipase Cγ (PLCγ), PI3K–AKT, Ras–Raf–MAPK, and STATs), resulting in an increased sensitivity to FGF and the promotion of tumor growth [32]. Researchers have detected mutations in the FGFR gene in ovarian and other gynecological tumors [33,34]. The association between the FGFR3 gene mutation and ovarian cancer has been the primary focus of research. Nevertheless, certain investigations that yielded similar findings to our own study also demonstrated the presence of FGFR3 mutations in borderline serous tumors, particularly those with invasive implants [35]. This provides encouragement for the development of targeted therapies to stop borderline serous tumors from progressing into LG serous tumors. Potential therapeutic drugs that target the FGFR3 gene are listed in Table 4.

The US Food and Drug Administration approved pazopanib (VotrientTM, GlaxoSmithKline) in October 2009 for the treatment of advanced renal cell carcinoma. It is an oral angiogenesis inhibitor that targets the VEGF receptor (VEGFR), the platelet-derived growth factor receptor (PDGFR), and c-Ki. Even though it has been suggested as a potential treatment for ovarian cancer, more recent research suggests that it is ineffective. The phase-III AGO-OVAR16 trial evaluating the use of pazopanib as a maintenance treatment for ovarian cancer showed a slight improvement in progression-free survival. However, it regrettably did not achieve the desired outcome of improving overall survival [36]. Debra L. reported that the combination of pazopanib and paclitaxel did not demonstrate superiority over paclitaxel alone in cases of resistant or recurrent ovarian cancer [37] Although this agent, which has undeniable antitumor activity, seems ineffective in advanced ovarian tumors, it may be a new treatment option to prevent the transition to LG serous tumors in borderline tumors.

Nintedanib is an oral tyrosine kinase inhibitor targeting VEGF receptor 1–3, FGFR 1–3, and PDGFR α and β. Progression-free survival in advanced ovarian cancer was found to be significantly improved when nintedanib was used in combination with carboplatin and paclitaxel in first-line treatment, albeit with more gastrointestinal side effects [38]. Single-agent nintedanib treatment has been shown to increase progression-free survival in patients with bevacizumab-resistant epithelial ovarian cancer. However, more research is required to confirm these findings [39].

Infigratinib is an orally bioavailable selective FGFR1-3 inhibitor. In clinical trials, infigratinib demonstrated disease control in 84% of patients diagnosed with advanced cholangiocarcinoma and in 64% of patients diagnosed with advanced urothelial carcinoma [40]. Jing Zhao’s work has demonstrated that infigratinib effectively inhibits the activation of the PI3K/AKT pathway and stimulates cell apoptosis. Consequently, it enhances the sensitivity of cisplatin-resistant ovarian cancer cells [34].

A few studies examined the relationship between ovarian cancer and dovitinib, AZD4547, ENMD, and brivanib, which are some other FGFR inhibitors that could be potential treatment agents (Table 4). More research needs to be done on efficiency [41,42,43,44].

The other differentially expressed gene between normal ovarian tissue and borderline serous tumors in our study, CD74, plays a crucial role in controlling the internal functioning of class-II MHC molecules and acts as a receptor for macrophage migration inhibitory factor (MIF). Cell proliferation, prostaglandin E2 synthesis, and extracellular signal-regulated kinase activation are some of the signaling processes triggered by MIF binding to CD74. The involvement of this ligand–receptor interaction in chronic inflammation and carcinogenesis has been reported. The expression of CD74 has been investigated in several types of cancer, but not ovarian cancer [45]. Hagemann reported that MIF expression increased in both borderline and malignant ovarian tumor cell lines compared to normal tissue. Considering the relationship between MIF and CD74, we can say that it is parallel to our findings [46].

Milatuzumab (hLL1, IMMU-115), is a humanized anti-CD74 monoclonal antibody. Preclinical research has shown that milatuzumab is effective against hematological malignancies [47]. Govindan S.V. reported that, although milatuzumab’s efficacy was high in the lymphoma model, it was also found to be effective in solid tumors when combined with SN-38 [48]. Additional research is required to assess the effectiveness of milatuzumab in solid tumors and ovarian cancer.

Integrin beta 2 (ITGB2) (CD18/LFA-1), a member of the leukocyte integrin family, is one of the differentially expressed genes between normal ovarian tissue and borderline serous tumors in our study. Integrins are a type of cell-surface protein involved in cell adhesion and cell-surface-mediated signal transduction. They play a critical role in the immune response by contributing to cell migration, proliferation, differentiation, and survival [49]. Research has shown that ITGB2 is highly expressed in a number of cancers, such as colorectal cancer, renal clear-cell carcinoma, papillary thyroid cancer, and breast cancer [50]. Many studies have demonstrated that ITGB2 is overexpressed in ovarian cancer relative to normal ovarian tissue; it is linked to metastasis and poor prognosis in ovarian cancer and can be used as a prognostic immunomarker [51]. Existing ovarian cancer studies suggest that ITGB2 targeted therapy should be explored. ITGB2-specific antibodies, potential target agents, have not yet been studied in solid tumors (Table 4). Their use in cases with inflammatory processes has been investigated, and their efficacy has been established [52]. To establish their efficacy in solid tumors, more research is required.

One of the most important differentially expressed genes between borderline and LG serous tumors is IGF-1, as mentioned in Table 4 part 2. The IGF pathway with its downstream effectors, which are PI3K/AKT/mTOR and RAF/MAP kinases, have well-defined roles as mitogens in carcinogenesis [53]. Its role in LG serous ovarian tumors was demonstrated in a study by King et al. [54]. They reported that the IGF-1 gene was upregulated and IGF-1 and IGF-1R were overexpressed in LG serous tumors with respect to borderline tumors, which is similar to our findings. Additionally, LG serous tumor cells were found to be more sensitive to IGF-1R inhibition than borderline cells. Dusigitumab, a human monoclonal antibody that binds to IGF-I/II, was studied in colorectal cancer cells, solid tumors, and sarcomas, revealing promising results [55,56,57].

ERBB4 is another upregulated gene in LG serous tumors compared to normal ovarian cells. The ERBB4 gene encodes an enzyme, receptor tyrosine-protein kinase erbB-4, which is a member of the epidermal growth factor receptor family [58]. Current evidence suggests that ErbB4 may act as a proto-oncogene, and it is primarily based on its association with other ErbB receptors. As yet, there is no strong evidence that either ERBB4 mutation or overexpression can induce cancer development and/or progression [59]. Many reports have highlighted the role of ERBB4 in cancer, and it is also related to the pathogenesis and prognosis of ovarian cancer [60,61]. Inhibitors of tyrosine kinases and blocking the activation of downstream pathways improved patient outcomes in various solid tumors [62]. Poziotinib, a panhuman epidermal growth factor receptor, was reported to decrease sphere formation, viability/proliferation, and induced G1 cell-cycle arrest and apoptosis in ovarian cancer stem cells [62,63]. In a study by Coleman et al., vandetanib, an oral tyrosine kinase inhibitor of VEGFR2/3, EGFR, and RET, was reported to have clinical activity as a single agent or in combination with taxane. However, vandetanib plus docetaxel combination failed to show a positive effect in women with progressive or refractory ovarian cancer [64]. This study did not provide detailed information on the percentage of LG serous tumors. Since ERBB4 is upregulated in LG serous tumors. The latter tyrosine kinase inhibitors and other drugs such as Osimertinib Mesylate, Dacomitinib, Afatinib Dimaleate, Ac-480, Gefitinib or Pelitinib that have not been studied in LG serous tumors but were shown to have clinical effects on other solid tumors [62] may be potential treatments for low serous ovarian cancer. Another orally administered small molecule inhibitor of BTK, which has also been reported to have off-target activity against the ERBB/EGFP family, ibrutinib, is an FDA-approved drug for hematological diseases [65,66,67]. Interestingly, in a case report by Gray et al., a significant clinical response was obtained by ibrutinib in LG serous tumors after organoid drug testing [68].

Another potential mechanism is related to the AR gene and its metabolism. This gene encodes a kind of nuclear receptor that is activated by the binding of androgenic hormones. In addition to its well-known physiological roles, it is also related to prostate, breast, and ovarian cancers [69]. In vitro studies suggested that AR activation is related to the induction of tumorigenesis and cancer progression as well as chemoresistance in ovarian cancer [70]. Antiandrogenic agents are commonly used for prostate cancer, but since the correlation was shown in preclinical studies in ovarian cancer, phase studies have been coming forward recently. In a phase-II trial, Geist et al. investigated the role of enzalutamide, in AR-positive recurrent high and low-grade serous ovarian cancer patients. The administered dose until disease progression or discontinuation was 160 mg of daily enzalutamide, and 6 months PFS was 19.8% for HG serous tumors and 38.5% for LG serous tumors [71].

Last but not least, the epithelial–mesenchymal transition (EMT) is the cornerstone in the metastasis of epithelial tumors. In our study, OXTR, SPP1, and PDGFRB genes found upregulated in LG serous tumors with regard to normal cells are related to EMT. OXTR (oxytocin receptor) has a well-known role in the mechanism of labor. This receptor was also shown to have roles in colon and ovarian cancer [72,73]. Ji et al. reported that oxytocin inhibits ovarian cancer cell metastasis by suppressing the expression of MMP-2 and VEGF [73]. In our study, OXTR was upregulated in LG serous tumors, contrary to the findings in the latter study. This conflict may be due to the types of ovarian cancer cells since Ji et al. studied SKOV3 cells, which is the model for HG serous tumors. SPP1 is another upregulated gene in our study related to the EMT mechanism. Wang et al. reported that SPP1 overexpression relates to T-cell exhaustion and a more aggressive phenotype in ovarian cancer [73,74]. In a similar study, Gao et al. emphasized that SPP1 expression is strongly correlated with tumor-infiltrating lymphocytes in ovarian cancer [75]. Thus, SPP1 expression may both be related to the metastasis mechanism and tumor microenvironment and may have a role in immunotherapies in ovarian cancer treatment. Table 5 contains information on additional differentially expressed genes (DEGs) and potential drugs that may be considered for future treatment of low-grade serous tumors.

Very little is known about the tumor microenvironment in LG serous tumors; one of those studies compared the lymphocyte infiltration in LG and HG serous tumors. Figure 6 shows immune-cell infiltration in benign, borderline, and LG serous tumor cells in our study. According to Ciucci et al., LG tumors exhibited a lower density of tumor-infiltrating CD68+ macrophage, along with an attenuated M2-skewed (CD163+) phenotype than benign and borderline tumors [76]. In another study by Li et al., M2 macrophage infiltration is strongly correlated with ITGB2 expression in ovarian cancer patients [51]. Similarly, SPP1, which is also an important DEG in our study, was reported to be significantly correlated with infiltrating levels of CD4+ T cells, CD8+ T cells, macrophages, neutrophils, and dendritic cells [75]. The correlation of the tumor microenvironment according to ITGB2 and SPP1 expressions is shown in Figure 7A,B.

Based on the performed bioinformatics analyses, the above-mentioned discussions provide insightful definitive conclusions. We promote to the readers the necessary molecular validation (in vivo/in vitro experiments). Taken together, the present study is a pioneering study in the field and can inspire remarkable various studies in the future.

## 5. Conclusions

This paper is one of the few studies that comprehensively discusses the genetic expression differences and their potential mechanisms in LG serous ovarian tumors in a biologic continuum from benign to borderline and malignant transformation. Since it is a rare type of epithelial ovarian tumor, its management is unsatisfactory, especially in advanced stages. This study provides a global view to investigate the specific targets and their downstream pathways. However, it is important to note that the current study is preliminary, and before considering these outcomes as fully reliable, subsequent in vitro and in vivo studies will be necessary to validate the results. Future research should focus on conducting targeted molecular and cellular studies to validate and expand upon the genetic targets and pathways identified here, offering the potential for more effective therapeutic strategies and improved management of LG serous ovarian tumors.

## Figures and Tables

**Table 1:** Data selected for the study from the GEO public functional genomics data repository.

Batch	Chip	GEO Series	Healthy Ovarian	Borderline	Low Serous	
1	GPL570	GSE18520	10			
2	GPL570	GSE27651	6	8	13	
3	GPL570	GSE14001	3		10	
4	GPL570	GSE27659			10	
5	GPL570	GSE73091			3	
6	GPL570	GSE9891		18		
7	GPL570	GSE14407	12			
8	GPL570	GSE36668	4	4		
9	GPL570	GSE54388	6			
		Total	41	30	36	107

**Table 2:** Tools and packages with their versions and the analyses performed using them.

	Package/Tool	Version	Aim	Reference
1	R	4.3.1	All analyses	[13]
2	Cytoscape	3.10.1	Pathway–gene–drug term figure plotting	[14]
3	Affy	1.80.0	Microarray gene-expression data Preprocessing	[15]
4	SVA	3.50.0	Batch-effect correction	[16]
5	M3C	1.24.0	UMAP plots	[17]
6	WGCNA	1.71–1	Coexpression network analysis	[7]
7	enrichR	3.2	Gene-set enrichment analysis	[8]
8	RCy3	2.22.1	Visualizing networks from Cytoscape R	[10]
9	rDGIdb	1.28.0	Drug–gene interaction analysis	[18]
10	immunedeconv	2.1.0	Estimation of tumor-microenvironment infiltration	[19]
11	ggpubr	0.6.0	‘ggplot2’-based publication ready plots	
11	GSCA		Genomics of Drug Sensitivity in Cancer	[12]

**Table 3:** The number of genes in the selected significant WGCNA modules.

Group	Modules	Number of Genes	Total Number of Genes in the Modules	Intersection with DEGs	Intersection Total
Healthy(−) vs. Borderline(+)	m1, m2, m9	51, 1476, 354	1881	29, 572, 28	629
Healthy(+) vs. Borderline(−)	m12, m13, m15, m16	1571, 92, 218, 2376	4257	384, 30, 72, 193	679
Borderline(−) vs. Low serous(+)	m11	839	839	120	120
Borderline(+) vs. Low serous(−)	m5, m8	44, 3486	3530	10, 390	400
Healthy(−) vs. Low serous(+)	m3, m4	4543, 2996	7539	640, 274	914
Healthy(+) vs. Low serous(−)	m12	1571	1571	562	562

**Table 4:** Pathway term gene–drug interaction results for genes in the intersection of modules and DEGs in healthy vs. borderline and borderline vs. low serous groups.

**Healthy vs. Borderline**				
**Pathway Type**	**Name**	**Gene**	**Drug**	**Effect Type**
KEGG	Rheumatoid arthritis	ITGB2	Mlnm-2201, Ame-133v, Erlizumab, Efalizumab, Rovelizumab, Lifitegrast	Inhibitory
	Cell-adhesion molecules			
	Staphylococcus aureus infection			
	Viral myocarditis			
GO-BP	Regulation of cell migration (GO:0030334)	CD74	Milatuzumab	Inhibitory
Hallmark	Estrogen response late	FGFR3	Derazantinib, Rabeprazole Sodium, Enmd-981693, Dovitinib, Pazopanib, Hydrochloride, Masitinib, Infigratinib, Hesperadin, Brivanib Alaninate, Ponatinib, Orantinib, Azd-4547, Enmd-2076, Cp-459632, Rogaratinib, Xl-228, Brivanib, Nintedanib Esylate, Xl-999, Nintedanib, Ly-2874455	Inhibitory
**Borderline vs. Low Serous**				
**Pathway Type**	**Name**	**Gene**	**Drug**	**Effect Type**
Hallmark	Xenobiotic Metabolism	IGF1	Dusigitumab	Inhibitory

**Table 5:** Pathway term gene and transcription factor gene–drug interaction results table for modules and DEGs intersection genes of healthy vs. low serous groups.

Healthy vs. Low Serous				
Pathway Type	Name	Gene	Drug	Effect Type
KEGG	Proteoglycans in cancer	ERBB4 (HER4)	Osimertinib Mesylate, Poziotinib, Vandetanib, Dacomitinib, Pelitinib, Afatinib Dimaleate, Ac-480, Gefitinib, Bms-690514, Jnj-26483327, Canertinib Dihydrochloride	Inhibitory
	Pathways in cancer	AR (TF gene)	Clascoterone, Apalutamide, Bicalutamide, Galeterone, Flutamide, Nilutamide, Enzalutamide, Darolutamide, Apc-100	Inhibitory
	ECM-receptor interaction	SPP1	Ask-8007	Inhibitory
	Proteoglycans in cancer	PRKACA	GSK-690693	Inhibitory
	Wnt signaling pathway			
	Pathways in cancer			
	Tight junction			
HALLMARK	Interferon Gamma Response	PSME2	Carfilzomib, Bortezomib	Inhibitory
	Interferon Alpha Response			
	Adipogenesis	PIM3	SGI-177, LGH-447, AZD-1208	Inhibitory
		DGAT1	Pradigastat	Inhibitory
	Hypoxia	PGF	Conbercept, Aflibercept	Inhibitory
	Bile Acid Metabolism	AR	Nilutamide, Galeterone, Flutamide, Enzalutamide, Darolutamide, Clascoterone, Bicalutamide, Apc-100, Apalutamide	Inhibitory
	Estrogen Response Early			
	Estrogen Response Late	SCNN1A	Triamterene, P-1037, Amiloride Hydrochloride	Inhibitory
	Estrogen Response Early			
	TNF-alpha Signaling via NF-kB	CCND1	Palbociclib, Briciclib	Inhibitory
	Estrogen Response Late			
	Estrogen Response Early			
	Apoptosis			
	Androgen Response			
	Apoptosis	HSPB1	Apatorsen	Inhibitory
	Complement	CLU	Custirsen Sodium, Custirsen	Inhibitory
	Coagulation			
	Cholesterol Homeostasis			
	Apoptosis			
	Cholesterol Homeostasis	FDPS	Zoledronic Acid, Risedronate Sodium, Pamidronate Disodium, Ibandronate Sodium	Inhibitory
	Epithelial–Mesenchymal Transition	OXTR	Retosiban, Relcovaptan, Epelsiban, Barusiban	Inhibitory
	KRAS Signaling Up	SPP1	Ask-8007	Inhibitory
	Epithelial–Mesenchymal Transition			
	Epithelial–Mesenchymal Transition	PDGFRB	Xl-999, Xl-820, X-82, Vatalanib, Tg100-801, Telatinib, Tandutinib, Tak-593, Sunitinib Malate, Sunitinib, Su-014813, Sorafenib Tosylate, Sorafenib, Rg-1530, Regorafenib, Quizartinib, Puquitinib, Pd-0166285, Pazopanib Hydrochloride, Pazopanib, Orantinib, Nintedanib Esylate, Nintedanib, Nilotinib, Motesanib, Midostaurin, Masitinib, Linifanib, Ji-101, Imatinib Mesylate, Imatinib, Ilorasertib, Hesperadin, Foretinib, Famitinib, Enmd-981693, Dovitinib, Dasatinib, Crenolanib, Cm-082, Cep-2563, Cediranib, Cdp-860, Axitinib, Anlotinib	Inhibitory
	Apoptosis			

## Data Availability

The microarray gene-expression datasets are available on the GEO database (Data access numbers are listed in Table 1).

## Supplementary Materials

The following supporting information can be downloaded at: https://www.mdpi.com/article/10.3390/cimb46030117/s1, Figures S1–S9: The results of the normalization of the datasets; Figure S10: Scale-free topology fit index curve; Figures S11–S20: Correlation analysis for gene expression and TME proportion. Excel sheets S1–S3: DEGs for each comparison, sheets S4–S11: Functional enrichment analysis results for each comparison group.
