# Supplementary material for: In Silico Approach to Molecular Profiling of the Transition from Ovarian Epithelial Cells to Low-Grade Serous Ovarian Tumors for Targeted Therapeutic Insights
Source: Curr Issues Mol Biol. 2024 Feb 26;46(3):1777–98. doi: 10.3390/cimb46030117 (PMC10968906; doi:10.3390/cimb46030117)
Supplement: Supplementary file 1 [file cimb-46-00117-s001.zip › cimb-2853245-supplementary.pdf]

Supplementary Materials

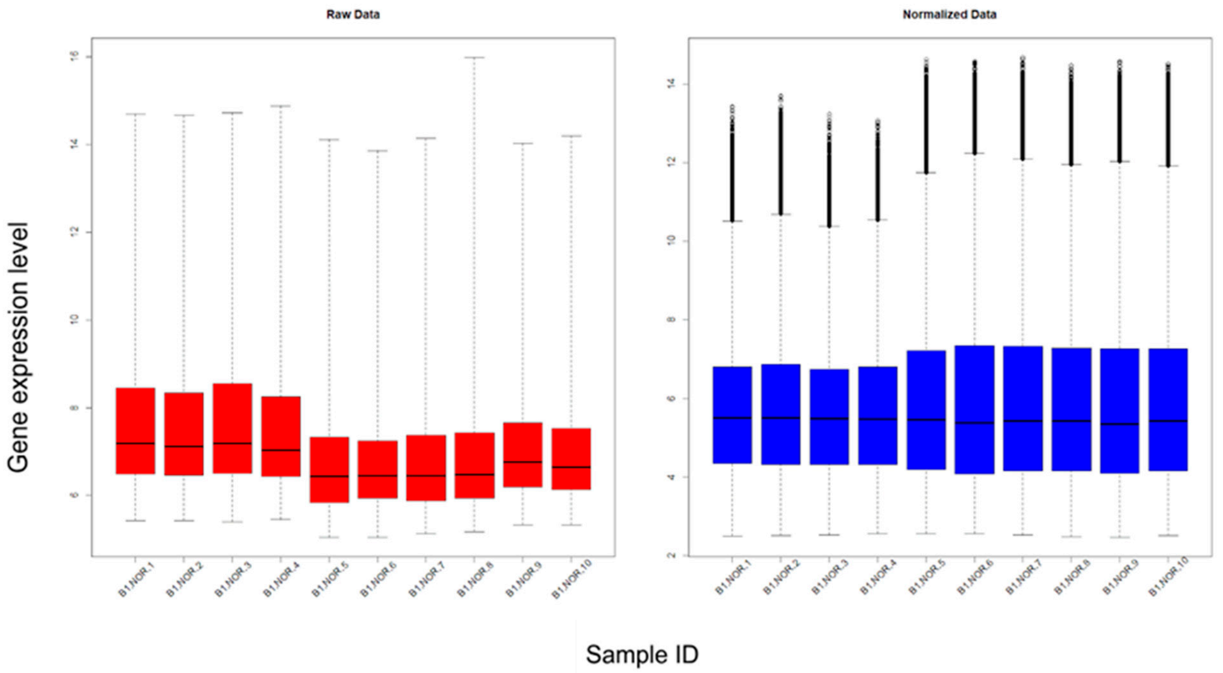

Figure S1. Boxplot of gene expression data of the GSE18520 dataset samples before (left) and after (right) normalization. (B1-> GSE18520, Nor->Normal Ovary, n=11).

https://www.ncbi.nlm.nih.gov/geo/query/acc.cgi?acc=GSE18520

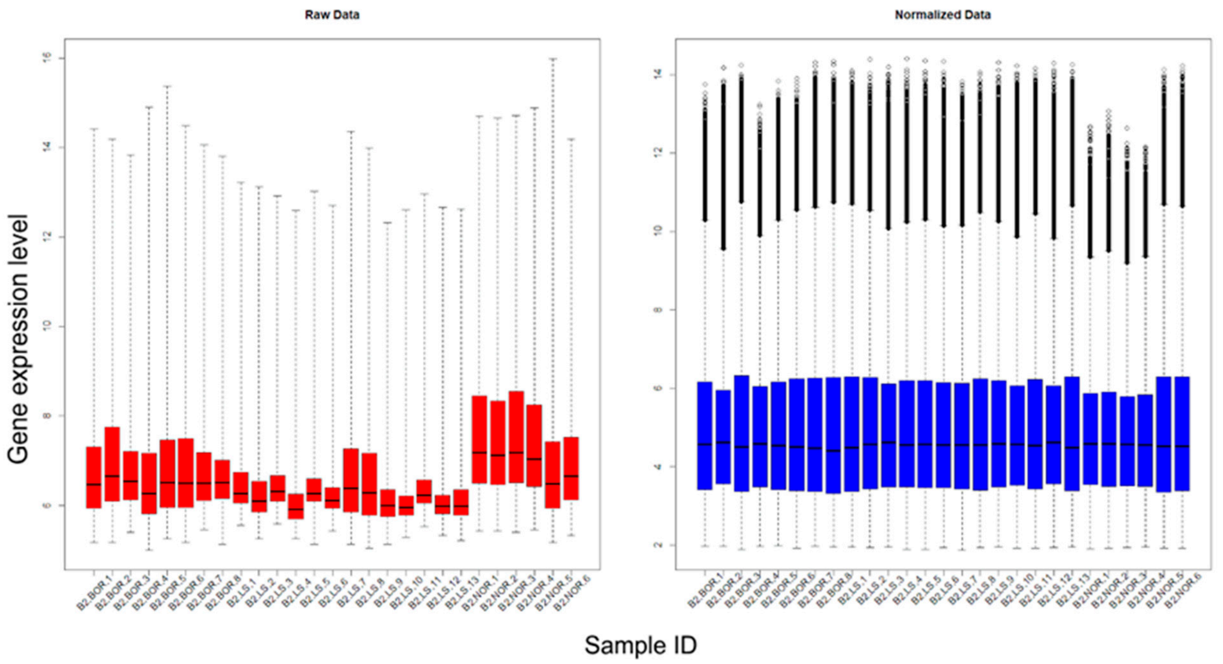

Figure S2. Boxplot of gene expression data of the GSE27651 dataset samples before (left) and after (right) normalization. (B2-> GSE27651, Nor->microdissected human ovarian surface epithelia (n=6), Bor->microdissected serous borderline ovarian tumors (LMP, n=8), LS->low-grade serous ovarian carcinomas (LGOSC, n=13)).

https://www.ncbi.nlm.nih.gov/geo/query/acc.cgi?acc=GSE27651

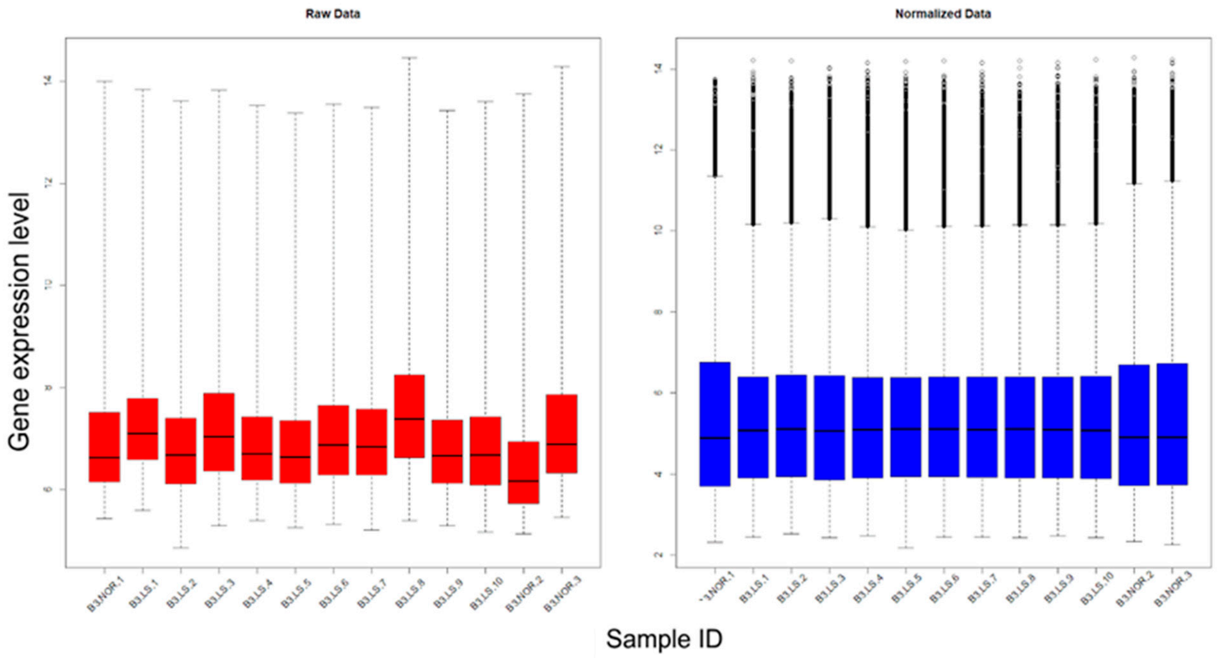

Figure S3. Boxplot of gene expression data of the GSE14001 dataset samples before (left) and after (right) normalization. (B3-> GSE14001, Nor->Normal human ovarian surface epithelia (n=3), LS->Low grade serous ovarian carcinoma (n=10)).
https://www.ncbi.nlm.nih.gov/geo/query/acc.cgi?acc=GSE14001

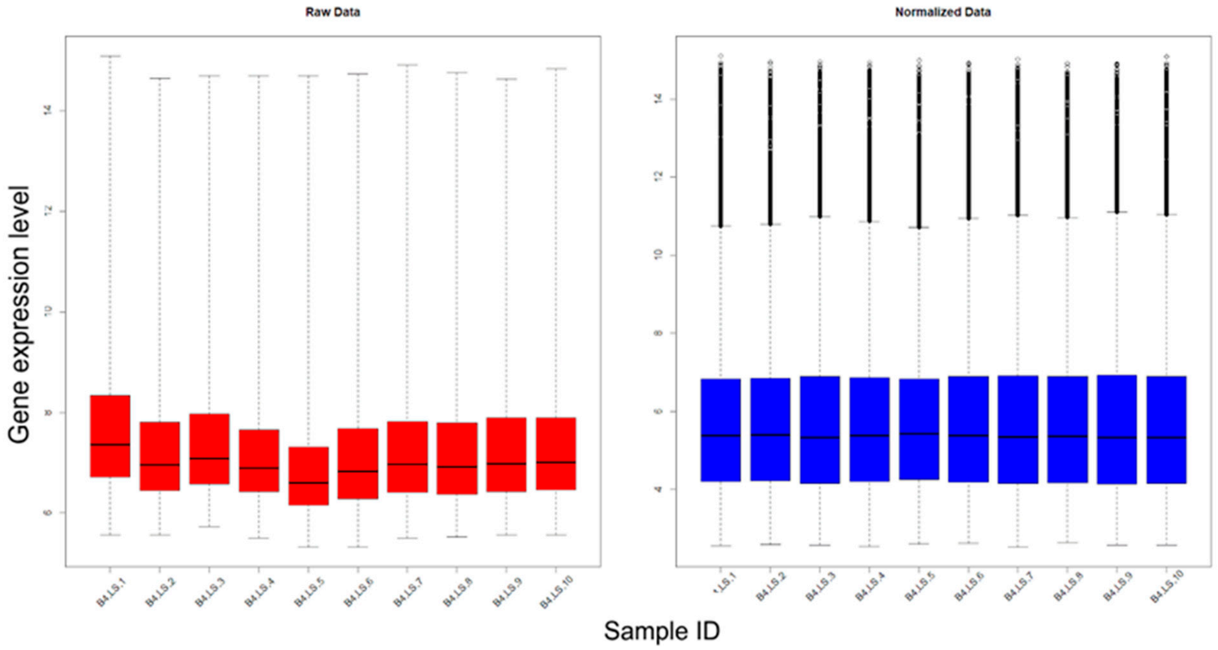

Figure S4. Boxplot of gene expression data of the GSE27659 dataset samples before (left) and after (right) normalization. (B4-> GSE27659, LS->Low-Grade Ovarian Serous Carcinomas (n=10)).
https://www.ncbi.nlm.nih.gov/geo/query/acc.cgi?acc=GSE27659

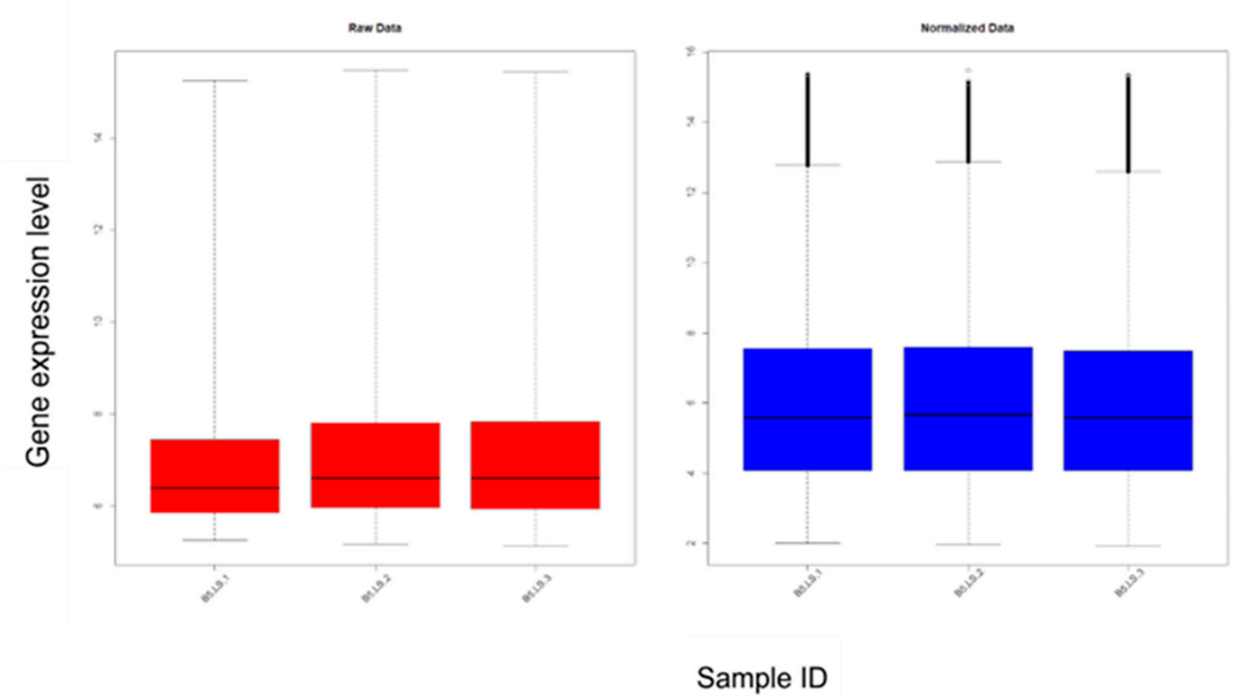

Figure S5. Boxplot of gene expression data of the GSE73091 dataset samples before (left) and after (right) normalization. (B5-> GSE73091, LS->low grade serous ovarian cancer, n=3).
https://www.ncbi.nlm.nih.gov/geo/query/acc.cgi?acc=GSE73091

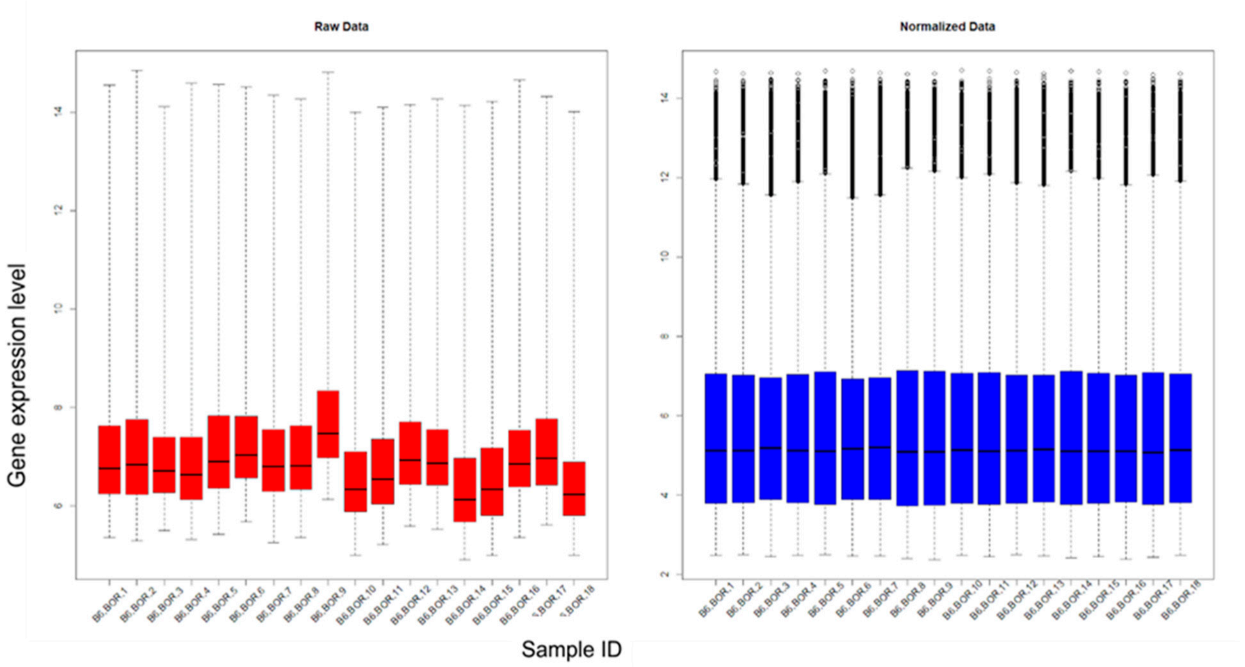

Figure S6. Boxplot of gene expression data of the GSE9899 dataset samples before (left) and after (right) normalization. (B6-> GSE9899, Bor->Borderline ovarian tumor samples).
https://www.ncbi.nlm.nih.gov/geo/query/acc.cgi?acc=GSE9899

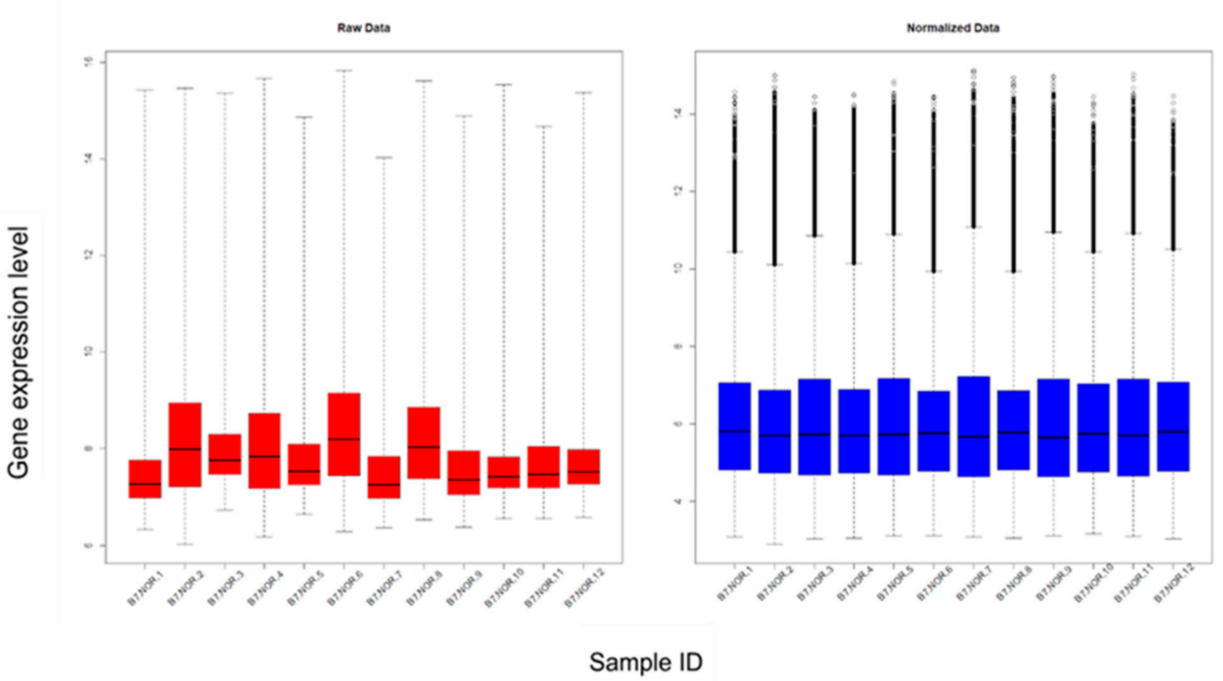

Figure S7. Boxplot of gene expression data of the GSE14407 dataset samples before (left) and after (right) normalization. (B7-> GSE14407, Nor->Healthy ovarian surface epithelial samples (n=12)).

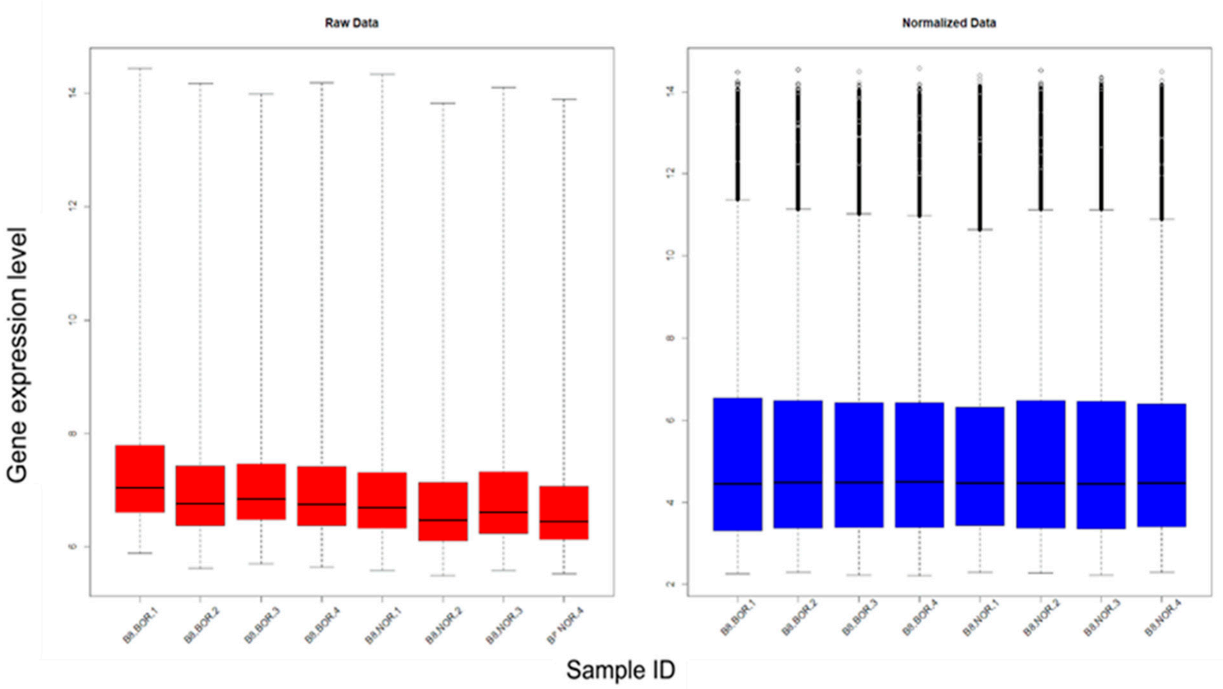

Figure S8. Boxplot of gene expression data of the GSE36668 dataset samples before (left) and after (right) normalization. (B8-> GSE36668, Nor->Superficial scraping from normal ovary (n=4), Bor->Serous ovarian borderline tumor (n=4)).

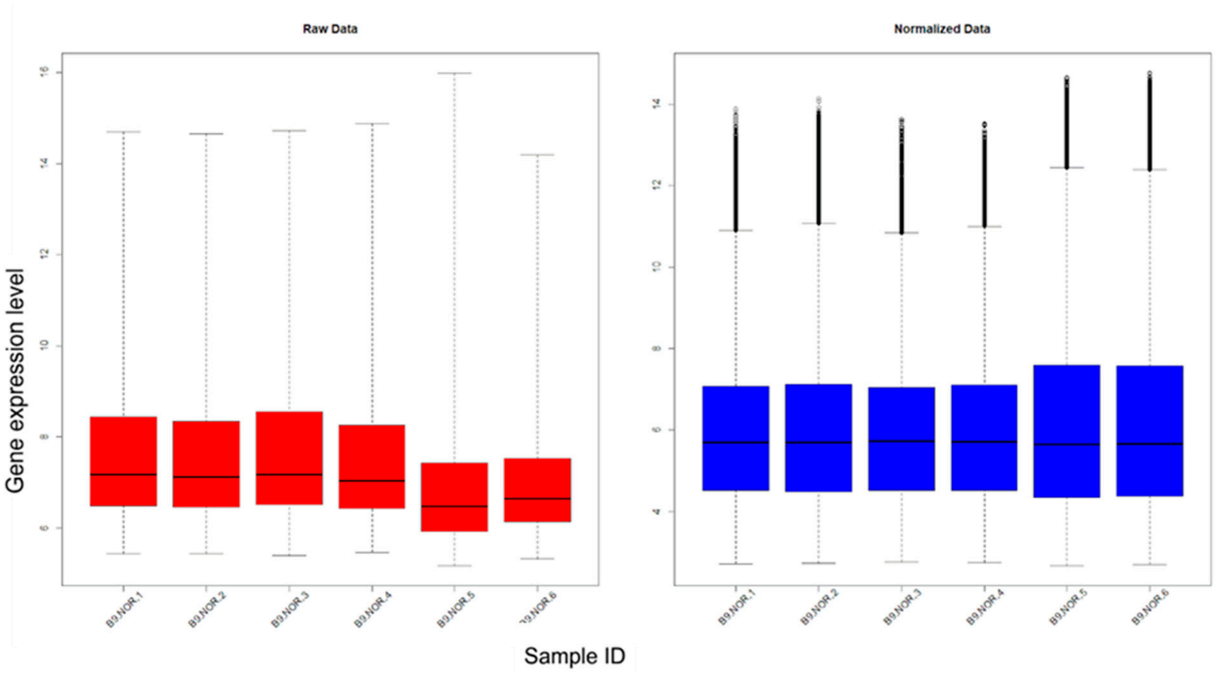

Figure S9. Boxplot of gene expression data of the GSE54388 dataset samples before (left) and after (right) normalization.(B9-> GSE54388, Nor->Human ovarian surface epithelium (n=6)).

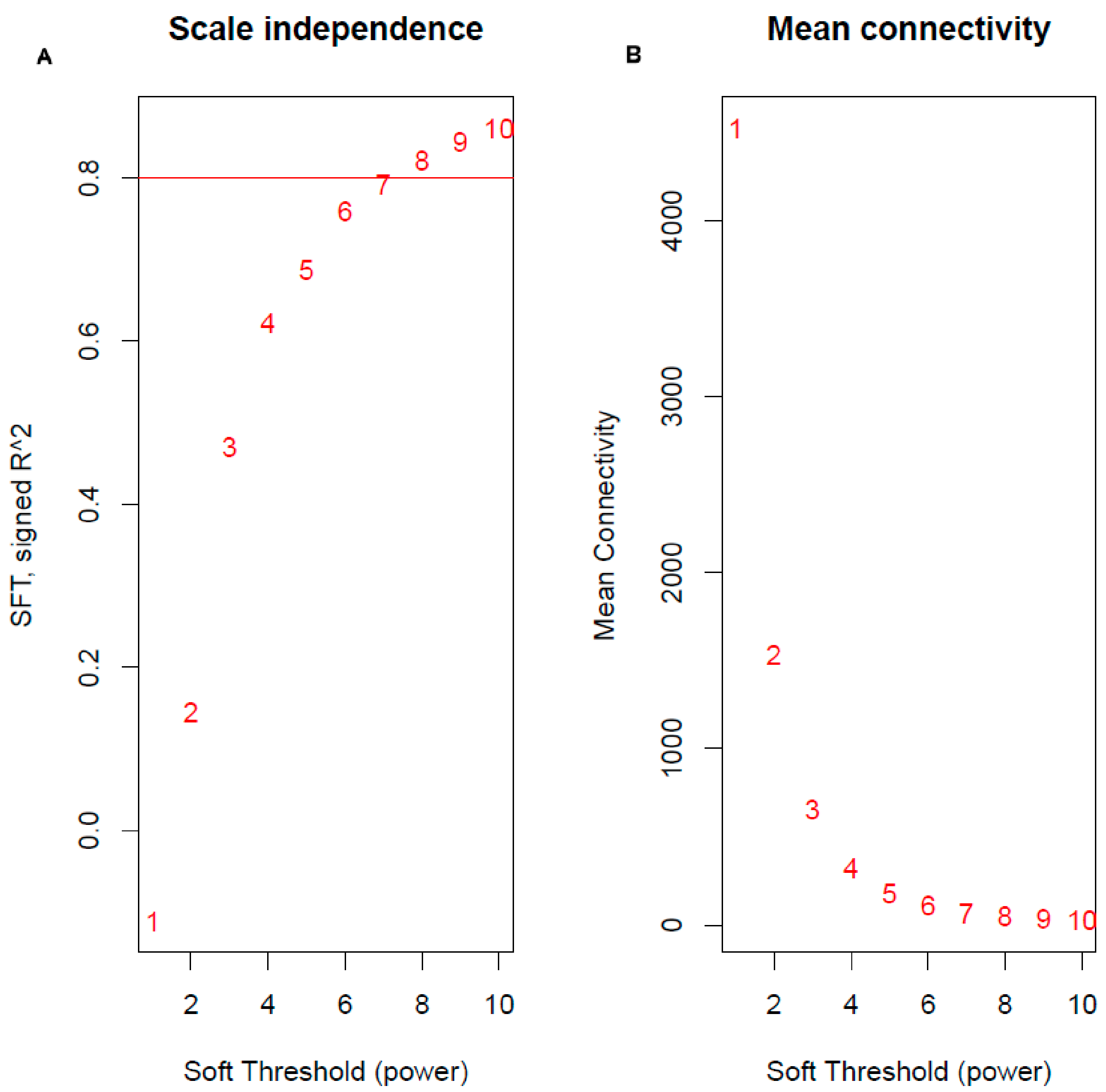

Figure S10. Sft shows how the structure of a network changes as the soft threshold is calibrated. The left and right panels show how well the network fits and how the average number of connections per node (average connectivity) changes a scale-free topology respectively as the soft threshold is increased.

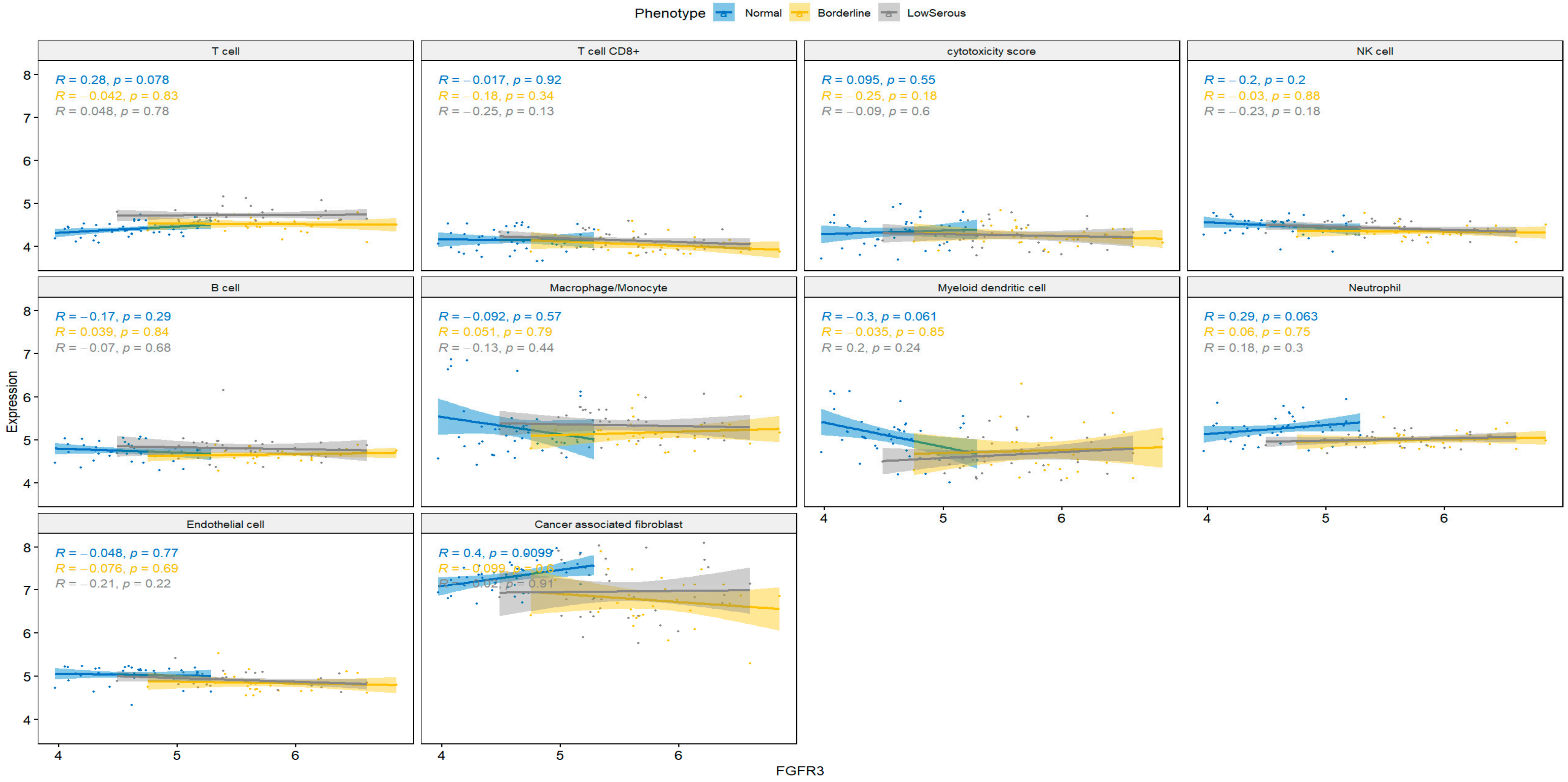

Figure S11. Correlation analysis for gene expression & TME proportion. For FGFR3, we found a moderate correlation between its expression and cancer associated fibroblast in normal tissue (r = 0.4, p = 0. 0099).

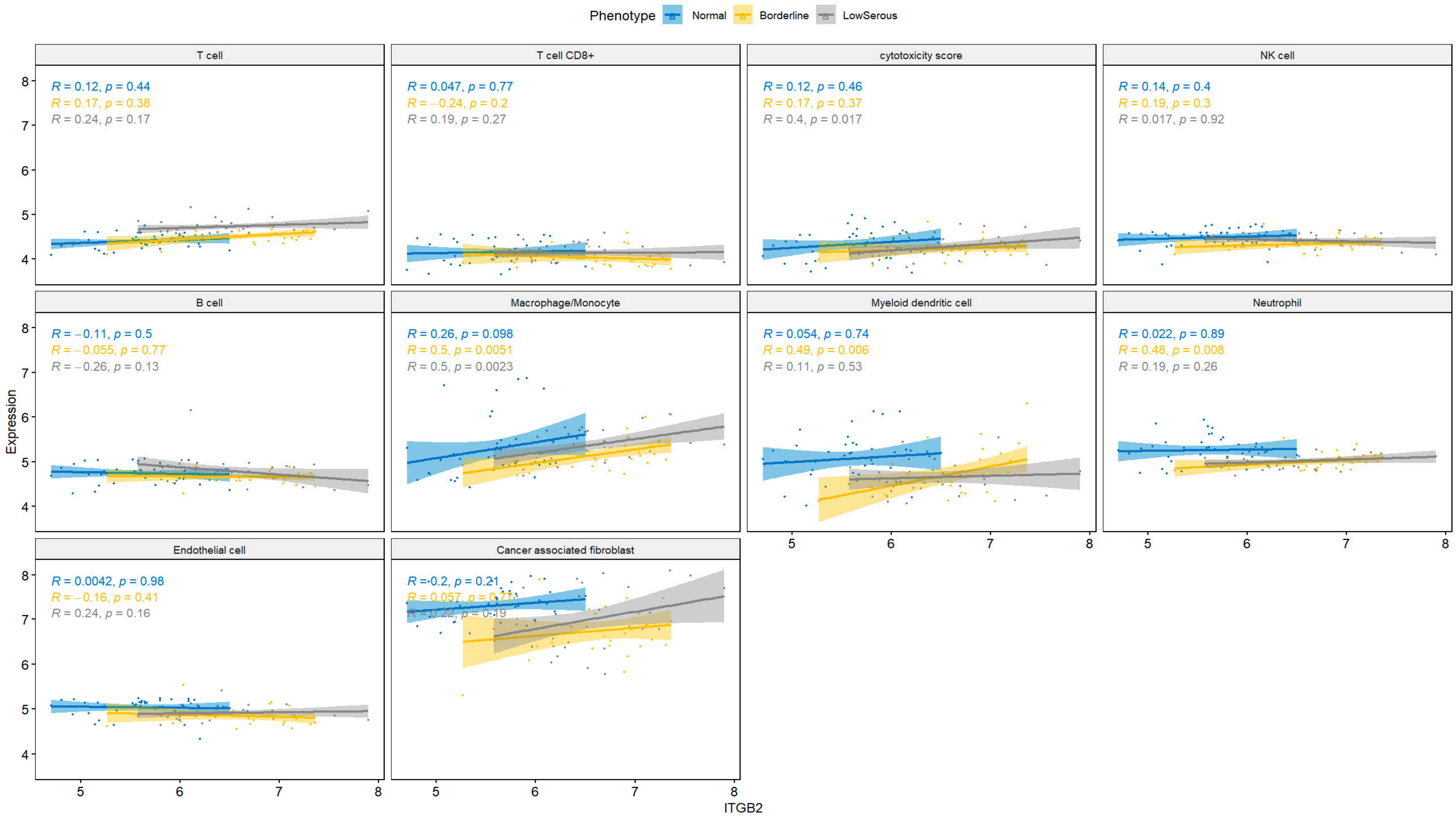

Figure S12. Correlation analysis for gene expression & TME proportion. For ITGB2, we found a moderate correlation between its expression and Macrophages/monocyte in borderline & low serous (r = 0.5, p = 0.0051 & r=0.5, p=0.0023)

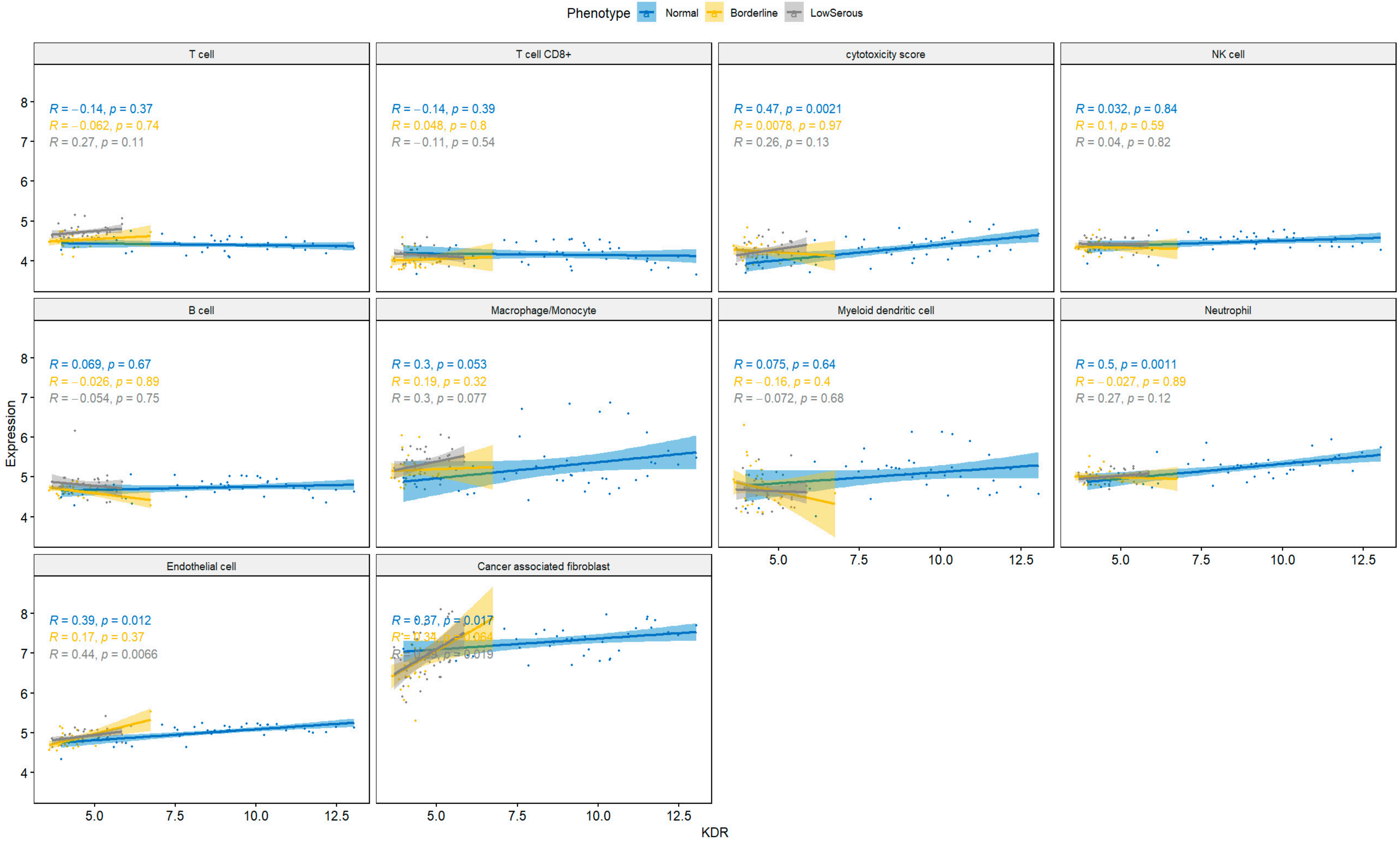

Figure S13. Correlation analysis for gene expression & TME proportion. For KDR, we found a moderate correlation between its expression and Endothelial cell in low serous tissue (r = 0.44, p = 0.0066).

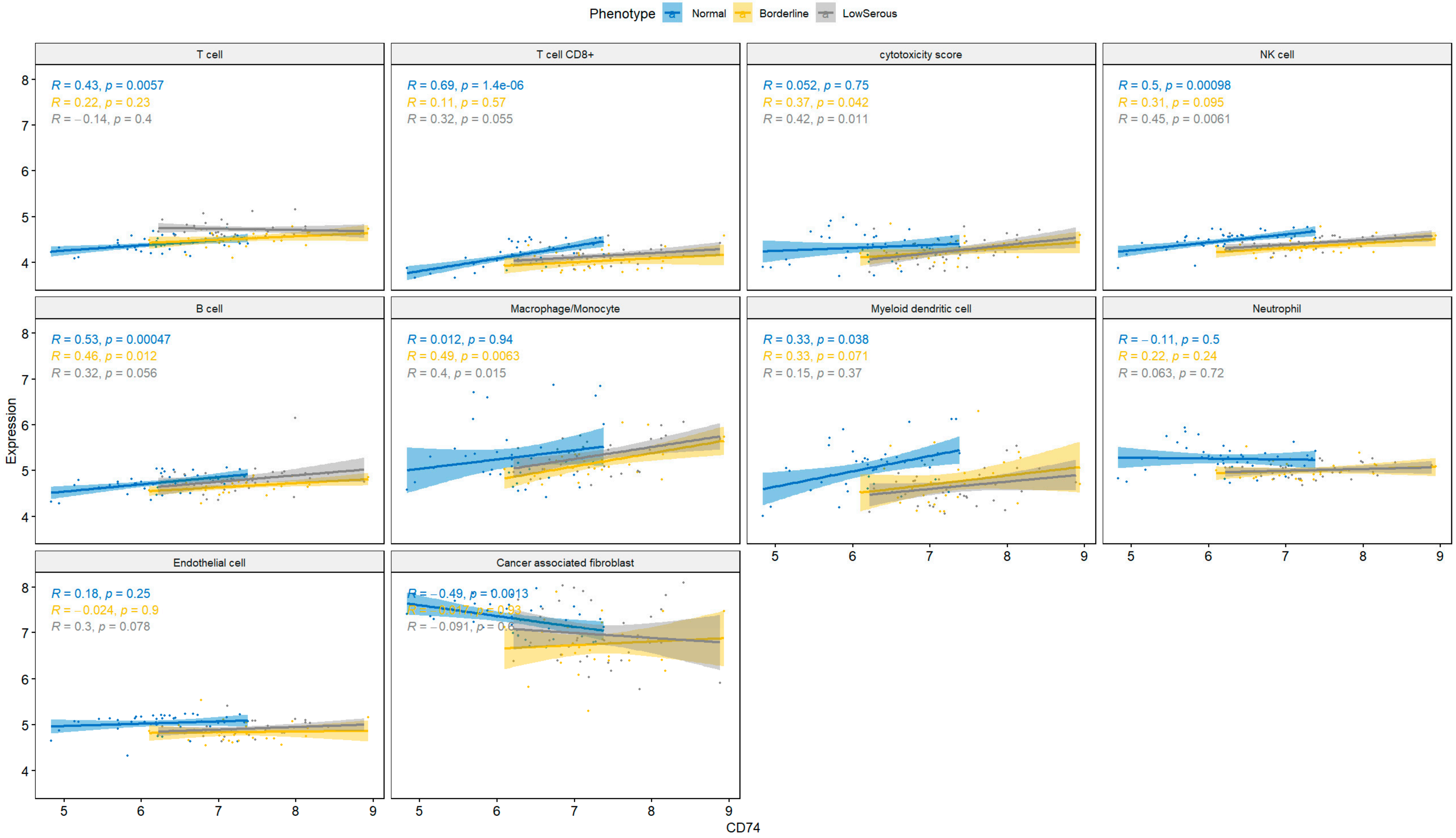

Figure S14. Correlation analysis for gene expression & TME proportion. For CD74, we found a moderate correlation between its expression and macrophages/monocyte in borderline tissue (r = 0.49, p = 0.0063).

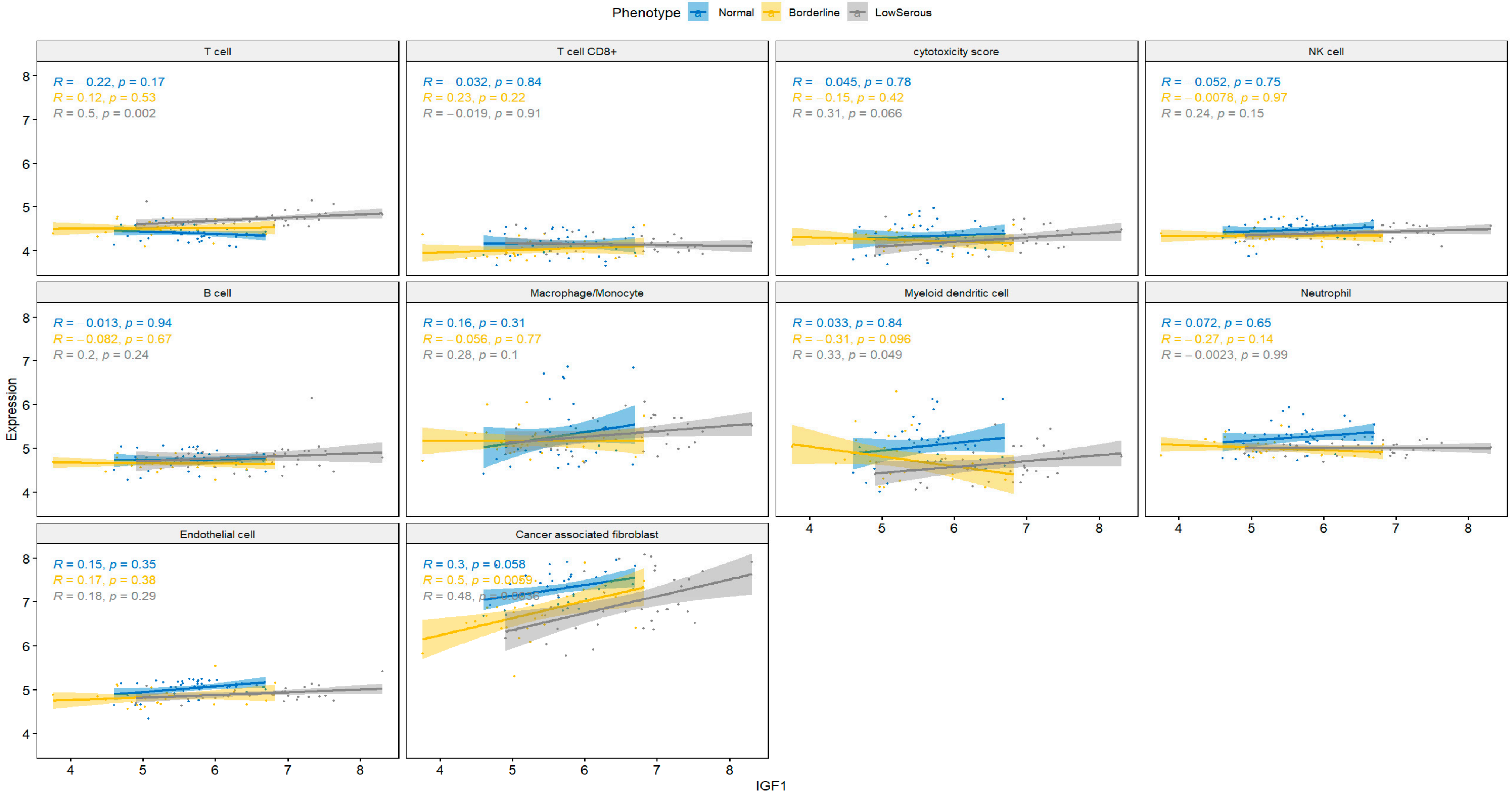

Figure S15. Correlation analysis for gene expression & TME proportion. For IGF1, we found a moderate positive correlation between its expression and borderline & cancer associated fibroblast in borderline tissue and low serous respectively (r = 0.50, p = 0. 0059, r =0.48, p=0.0036).

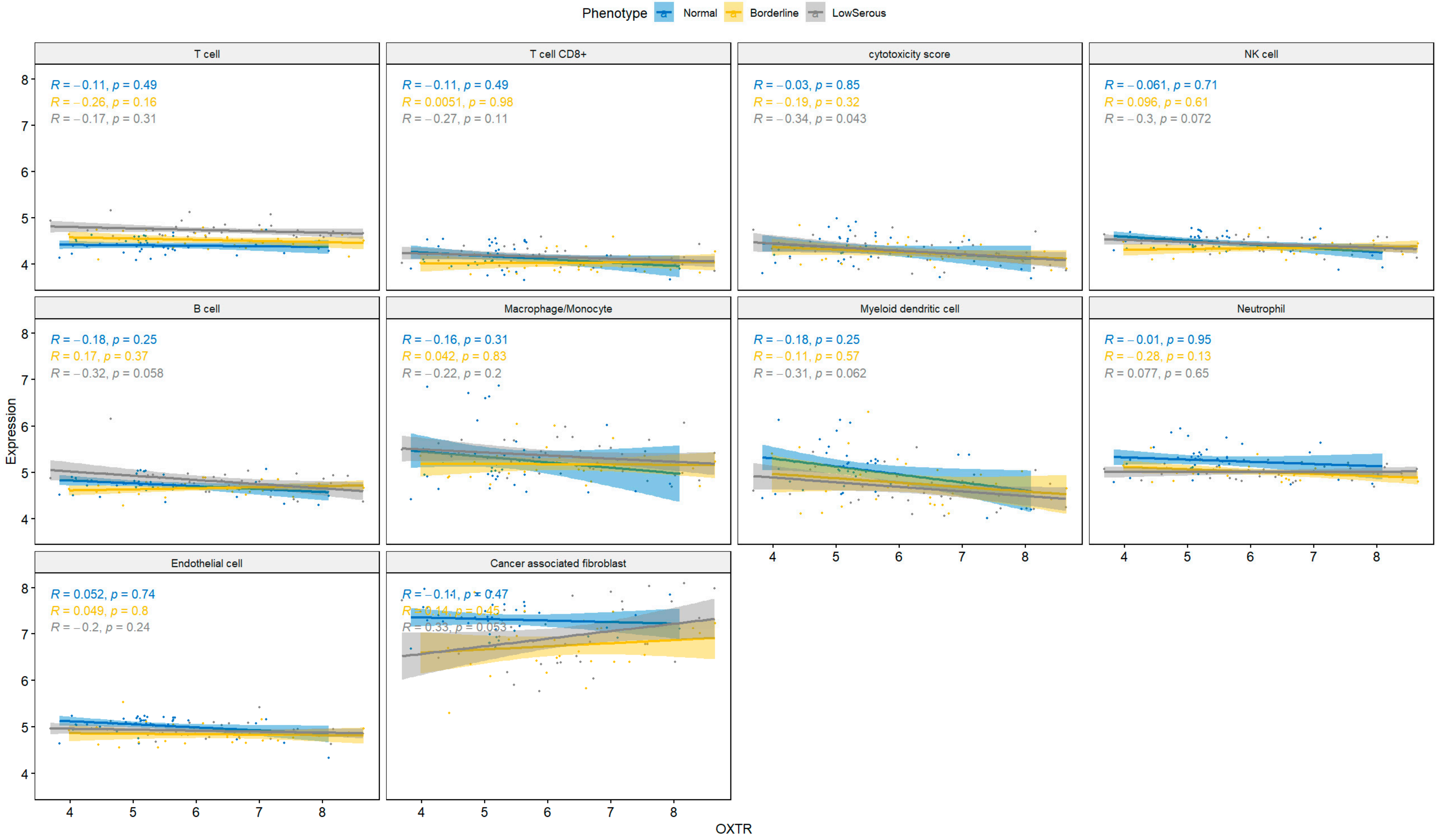

Figure S16. Correlation analysis for gene expression & TME proportion. For OXTR, we found moderate negative correlation expression between cytotoxicity score in low seous (r = -0.34, p = 0.043).

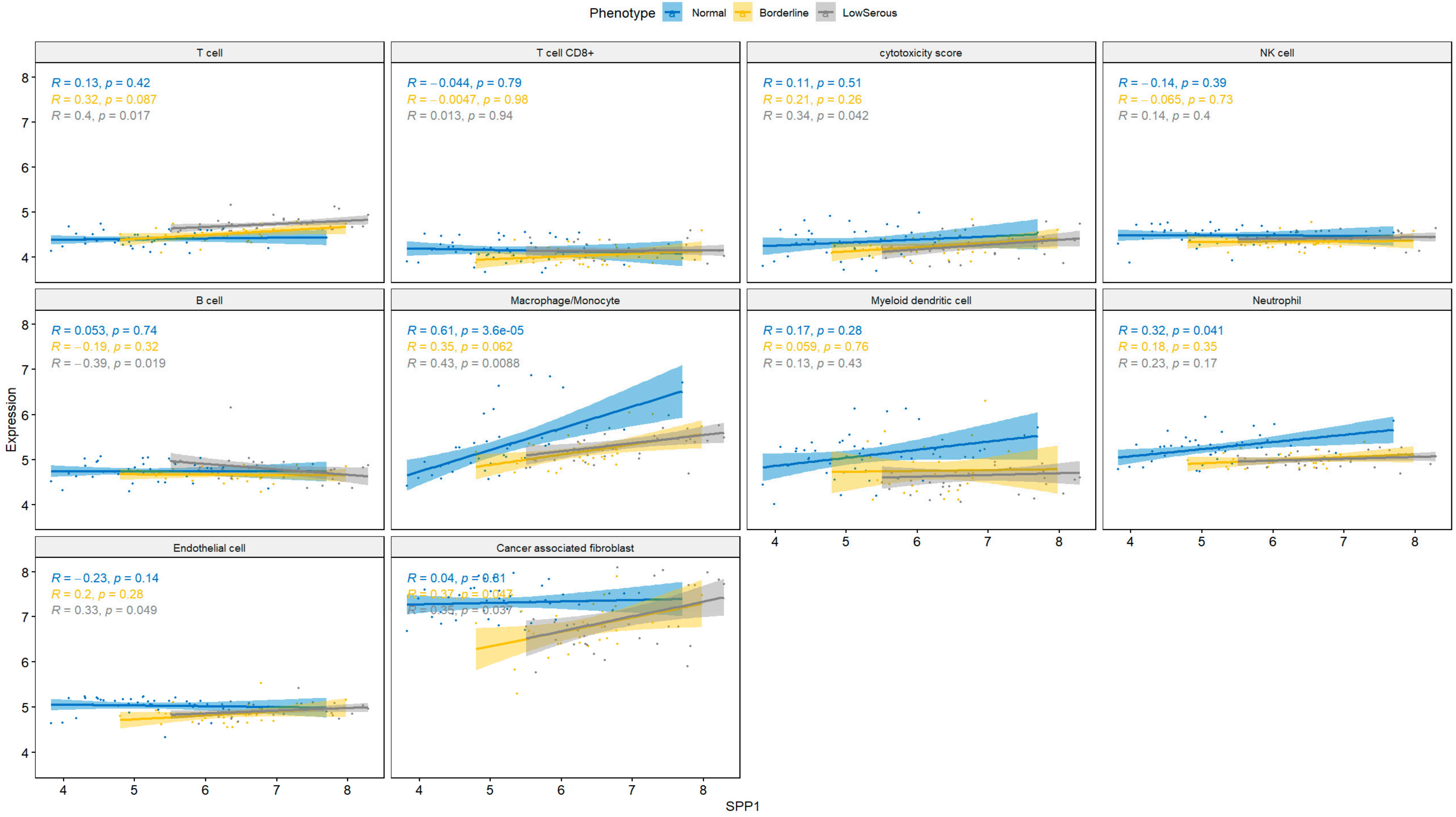

Figure S17. Correlation analysis for gene expression & TME proportion. For SPP1, we found moderate positive correlation expression between macrophages/monocyte in low serous tissue (r = 0.43, p = 0.0088).

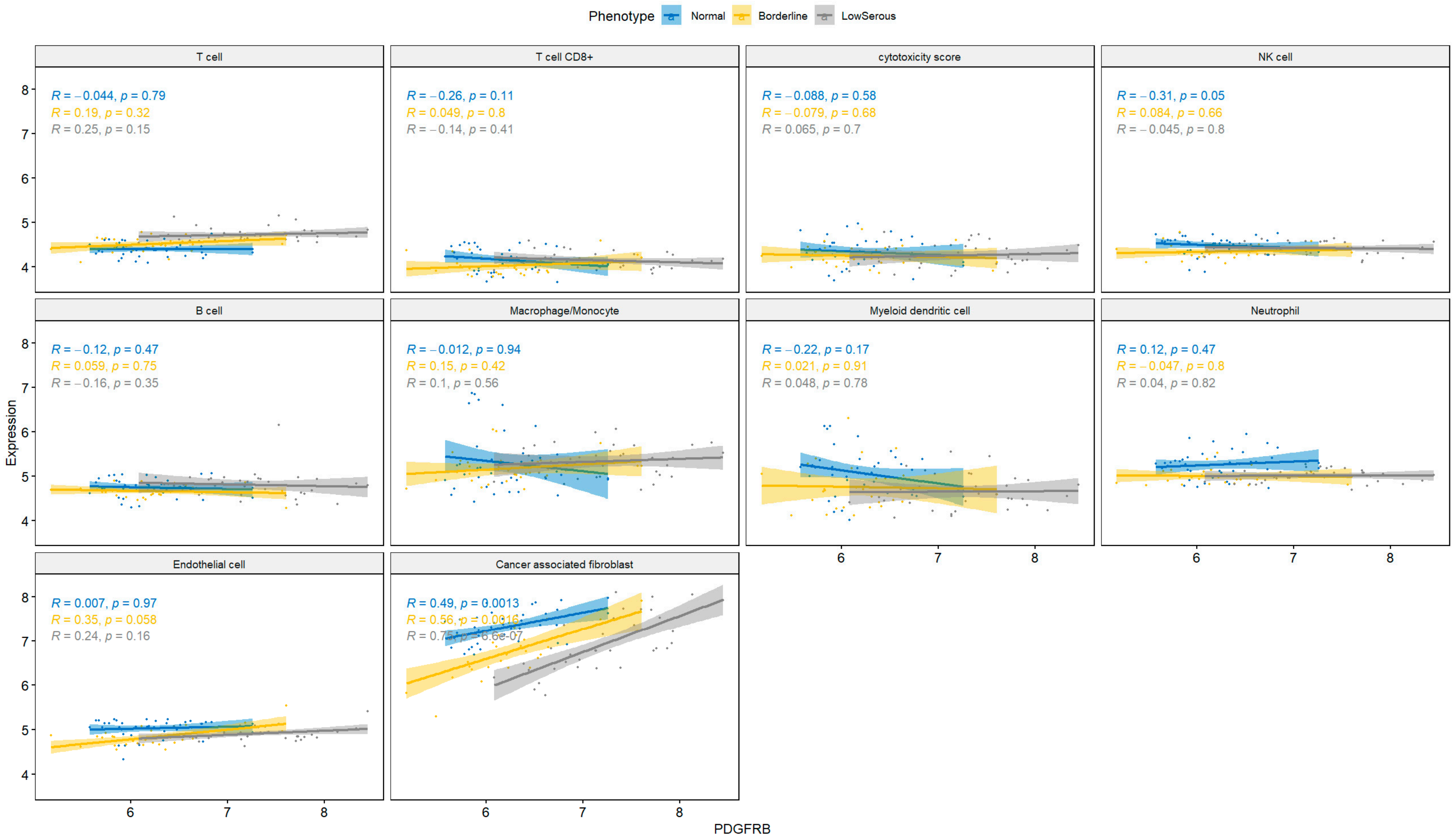

Figure S18. Correlation analysis for gene expression & TME proportion. For PDGFRB, we found high positive correlation expression between Cancer assocpated fibroblast in low serous tissue (r = 0.75, p = 6.6e-07).

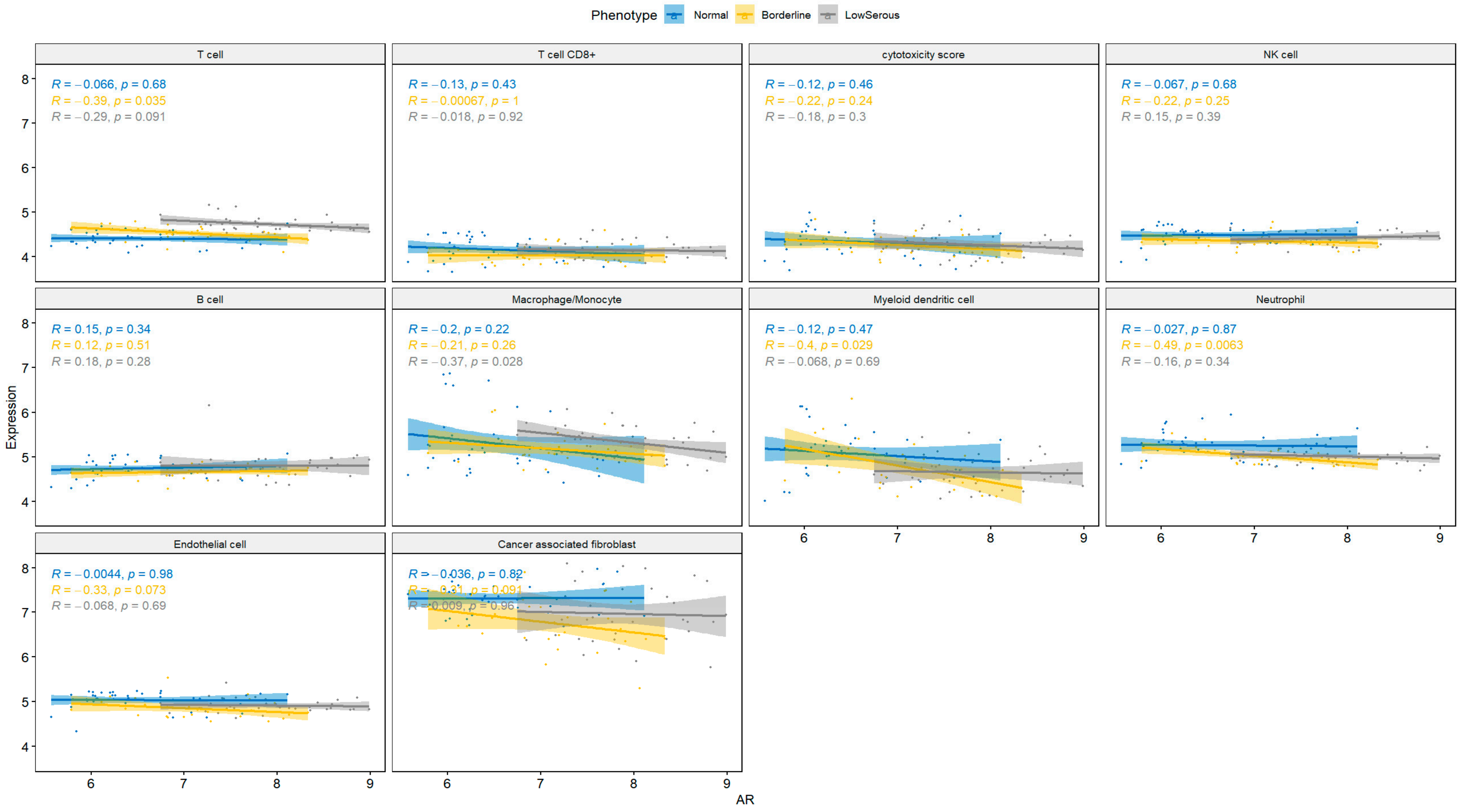

Figure S19. Correlation analysis for gene expression & TME proportion. For AR, we found moderate negative correlation expression between Neutrophil in borderline tissue (r = -0.49, p = 0.0063).

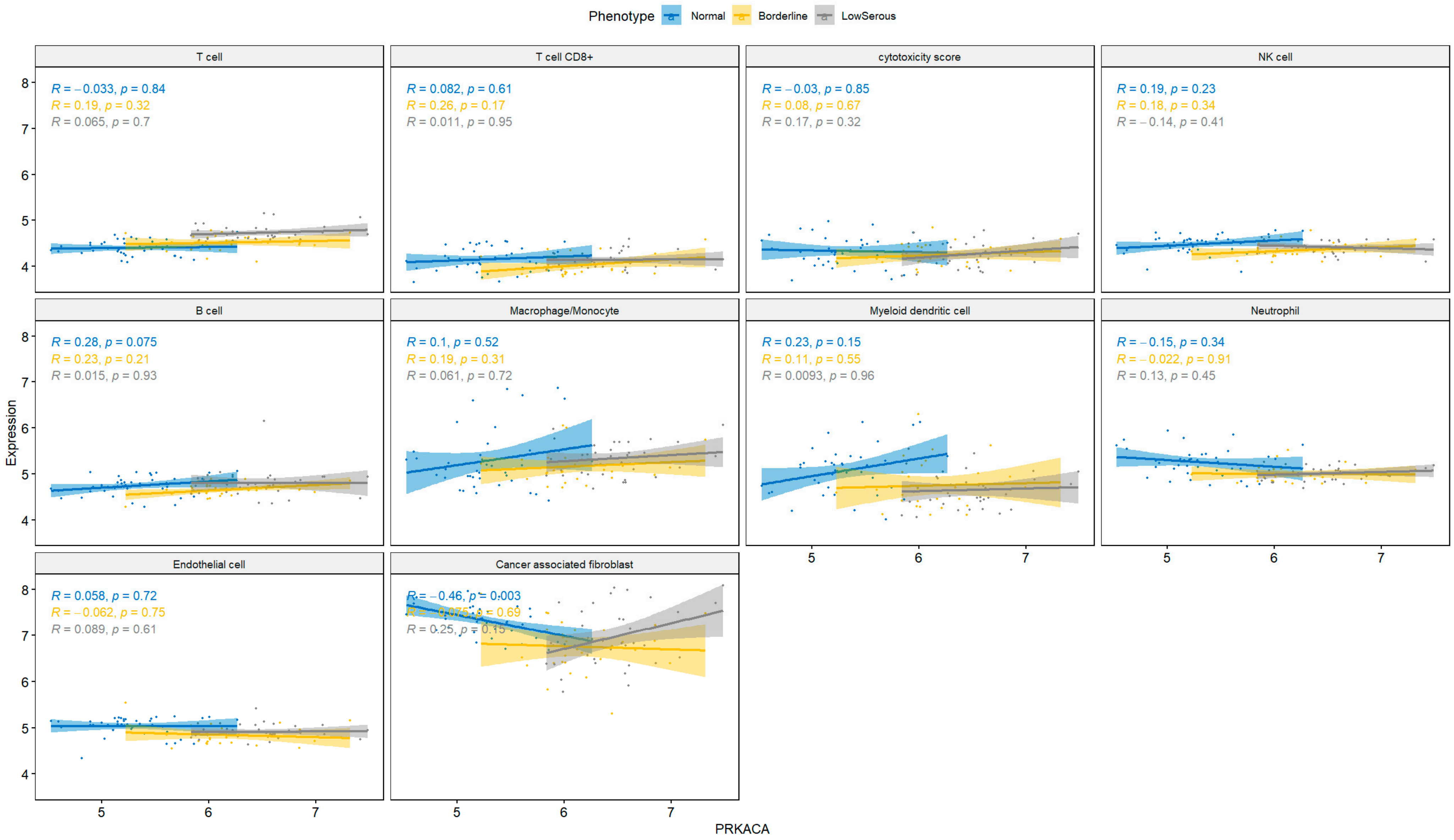

Figure S20. Correlation analysis for gene expression & TME proportion. For PRKACA, we found moderate negative correlation expression between Cancer associated fibroblast in normal tissue (r = -0.46, p = 0.003).
